# Agri-Food Surplus, Waste and Loss as Sustainable Biobased Ingredients: A Review

**DOI:** 10.3390/molecules27165200

**Published:** 2022-08-15

**Authors:** Joana P. B. Rodrigues, Ângela Liberal, Spyridon A. Petropoulos, Isabel C. F. R. Ferreira, Maria Beatriz P. P. Oliveira, Ângela Fernandes, Lillian Barros

**Affiliations:** 1 Centro de Investigação de Montanha (CIMO), Instituto Politécnico de Bragança, Campus de Santa Apolónia, 5300-253 Bragança, Portugal; 2 Laboratory of Vegetable Production, Department of Agriculture, Crop Production and Rural Environment, University of Thessaly, Fytokou Street, N. Ionia, 384 46 Volos, Greece; 3 REQUIMTE/Department of Chemical Sciences, Faculty of Pharmacy, University of Porto, Rua Jorge Viterbo Ferreira no. 228, 4050-313 Porto, Portugal

**Keywords:** circular economy, Innovative products, surplus, waste and loss, sustainable agriculture, vegetable

## Abstract

Ensuring a sustainable supply of food for the world’s fast growing population is a major challenge in today’s economy, as modern lifestyle and increasing consumer concern with maintaining a balanced and nutritious diet is an important challenge for the agricultural sector worldwide. This market niche for healthier products, especially fruits and vegetables, has increased their production, consequently resulting in increased amounts of agri-food surplus, waste, and loss (SWL) generated during crop production, transportation, storage, and processing. Although many of these materials are not utilized, negatively affecting the environmental, economic, and social segments, they are a rich source of valuable compounds that could be used for different purposes, thus preventing the losses of natural resources and boosting a circular economy. This review aimed to give insights on the efficient management of agri-food SWL, considering conventional and emerging recovery and reuse techniques. Particularly, we explored and summarized the chemical composition of three worldwide cultivated and consumed vegetables (carrots, broccoli and lettuce) and evaluate the potential of their residues as a sustainable alternative for extracting value-added ingredients for the development of new biodynamic products.

## 1. Introduction

Over the years, food has been one of the main concerns of man, as the methods of search, production and distribution undergoes frequent changes, being closely linked to the way humans eat [1,2]. In addition to its importance in satisfying basic needs, food is the key factor in preserving human health [3,4], as the adoption of healthy eating habits is highly reflected in the acquisition of essential nutrients, which reduce the risk of developing certain diseases, improving longevity and promoting optimal physical and mental well-being [5].

Agriculture is one of the main activities practiced around the world and one of the main economic processes on which the general prosperity of civilization is based [6,7]. In parallel, all over the world the increasing scientific and technological development, alongside the exponential growth of the human population, create a large gap between the demand and supply of food. With the advent of modern civilization and industrialization, agriculture began to be commercialized and intensified on a larger scale, resulting in the generation of huge amounts of agri-food SWL, putting the entire environmental balance under threat [8]. However, modern agriculture must be able to address a wide range of challenges by promoting environmental conservation and the effective management of natural resources, ensuring food security and sustainability. Consequently, in recent years, researchers have been dedicated to the development of approaches that enable the integrated management of agri-food SWL, preventing them from accumulating in nature and, consequently, negatively affecting the environment.

Agriculture SWL are one of the most abundant and renewable sources of compounds of interest on the planet (e.g., oils, proteins, fibers, phenolics and other bioactive compounds) which have the potential to be used in secondary processes, serving as food, feed, fuel, and/or as a source of a wide range of chemicals [7,8,9], thus being identified as number one priority for the application of European Circular Economic Strategy by the European Commission [10]. According to FAO [11], about a third of the food produced worldwide is lost or wasted every year, with about 22% of this loss consisting of agri-food SWL from fruits and vegetables. These residues are responsible for 8 to 10% of greenhouse gas emissions, 23% of fertilizer consumption, 25% of fresh water used in agriculture and for the use of arable land [6,12].

In this review, the foundations of sustainable agriculture and the efficient management and recovery of SWL derived from this activity will be explored, emphasizing their reuse for the development of new biodynamic products/formulations. In particular, the potential functionalities of agri-food SWL obtained from some of the most consumed vegetables in the world, namely carrots (*Daucus carota* L.), broccoli (*Brassica oleracea* L.) and lettuce (*Lactuca sativa* l.), will be explored, as well as their composition in bioactive compounds, with the ultimate goal of providing information for the development of new bio-based products and promoting the circular economy concept.

## 2. Food Security

The use of the term food security began after the end of World War I, a period in which food was a powerful weapon against countries/communities with insufficient capacity to produce their own food [13]. Its application assures the safe consumption of food within the scope of public health, suggesting its nature free from chemical, biological, and physical substances that may endanger human health. On the other hand, this term guarantees the availability, accessibility, and proper use of food, encompassing the entire set of needs to obtain a nutritious and balanced diet [14,15,16].

Currently, food security is often threatened due to the shortage of natural resources, population growth, volatile food prices, restraints in consumption habits, climate change, and food loss and waste [16]. Despite the sharp increase in food production resulting from the exponential increase in the world’s population, about one in nine people is food insecure [17]. Therefore, it has become a high priority to meet the growing world agricultural demand in a sustainable way, an objective being defined as the “Grand Challenge” of food security [18], which recognizes that sustainability, especially at the environmental level, must be considered as an explicit dimension of food security to ensure the availability, accessibility, use and stability of food [19]. Thus, food safety programs must provide effective quality control of the entire food chain, from production, storage, distribution to consumption of fresh or processed foods, as well as the necessary handling processes [20].

Recently, people have become more aware of the extent of food loss and waste along the entire food supply chain, while the reduction of these residues represents an essential measure within the structure of the sustainable food system to face the Grand Challenge of food security [19]. Now, and as part of the food security solution, it is well established that the use of advances in the agriculture sector, combined with the reduction of agri-food SWL and changes in daily eating habits, can double agricultural production and reducing environmental impacts [18]. One way to increase food safety without compromising the environmental burden of the agricultural process is to reduce agri-food SWL from farm to consumer. Here, the recovery of wasted food presents a great opportunity to reduce production demand, given that around 1.6 billion tons of food are wasted every year [21]. In this field, food science and technology play a leading role in improving food and nutrition security through the development of technologies capable of the preservation and stabilization of food products which, along with other features, allow the extension of its shelf life. Some of the agri-food SWL and secondary processing streams are directed to different uses, such as animal feed, chemical production, composting and energy, or are just dumped into landfills. However, the preferred strategy to improve food security is performed through the valorization and recovery of agri-food SWL, and its use as food, feed, fuel, and others [22], thus preventing their accumulation and consequent harmful effects on the environment.

## 3. Agriculture and Food Sustainability

Currently, the establishment of an agricultural sector capable of continuously supplying food and other essential resources for a world population in constant growth is of critical importance for the existence and preservation of any human activity. However, the ability of agriculture to meet the current and future human needs is threatened by a number of issues that include climate change, loss of biodiversity, soil degradation and pollution, rising production costs and poverty, among others [23,24]. 

According to the United Nations [25], agriculture production is expected to reach 8.5 billion people by 2030, rising further to 9.7 billion by 2050. However, intensive agriculture is associated with several problems such as the depletion of non-renewable natural sources, soil damage, adverse effects of agricultural chemicals on human health and the environment, and poor food quality [26,27,28]. At the same time, the availability of arable land also becomes a major issue, with the demand of greater volumes of production in shorter periods of time, resulting in serious problems of global pollution [29]. Thus, the implementation of sustainable approaches becomes of vital significance, with the ultimate purpose of addressing climate adaptation and mitigation, reduction of greenhouse gas emissions, prevention of natural disasters and maintenance of soil health [30]. Here, sustainable agriculture is presented as an ecosystem-oriented approach that involves the use of biological resources to increase production, avoiding the risk of pests and diseases, and taking into account the base of natural resources and their conservation [6].

**Figure 1:**
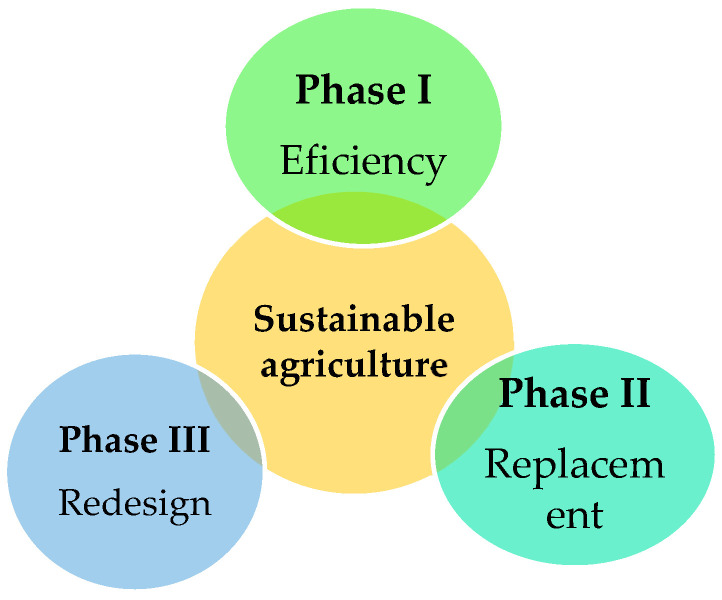
Transactional phases for sustainability in the agricultural environment.

According to FAO, sustainable agriculture is defined as a system that improves the efficiency of the use of natural resources, while preserving, protecting and improving natural ecosystems, sustaining rural livelihoods and social well-being, and increasing the resilience of people, communities and ecosystems [31]. One of the main challenges agriculture is facing around the world is reconciling growing food production with more sustainable agricultural practices. The growing concern about the harmful effects resulting from increased production has promoted changes in the paradigm of how agricultural systems can be used more efficiently, both in food production and in reducing environmental impacts, translating into many calls for a more sustainable agriculture [32]. Three transactional phases for sustainability in the agricultural environment have been proposed (Figure 1), among them efficiency, replacement and redesign. The first two, while crucial, are not sufficient for maximizing the co-production of favorable agricultural and environmental outcomes [33]. In the efficiency stage, a more sustainable use of existing agricultural resources is promoted, since many of these are wasted, culminating in the degradation of the farm’s natural capital or the flight of agrochemical products, which entails increased costs for the said farm and associated branches [33]. An example of this is post-harvest losses, which reduce the availability of food, directly contributing to the loss of efficiency and income generated by other means. Rationalizing the use of fertilizers, pesticides and water, on the other hand, promotes efficiency gains on the farm, causing less impact on the environment and human health [34,35,36]. In the replacement phase, the development of new crop varieties and livestock species allows for the replacement of less efficient components of the system with more efficient ones, such as certain plant varieties with greater capacity to convert nutrients into biomass, that are drought tolerant and/or resistant to salinity changes and are resistant to specific pests and diseases. Other substitution strategies focus on the use of biological control agents to the detriment of synthetic agrochemicals [1]. The third phase, the redesign, is fundamental for achieving sustainability on a larger scale, given that the redesign of ecosystems is essential in taking advantage of ecological processes, such as predation, parasitism, N fixation, and trophic dependencies, among others [37,38]. At this stage, the main objectives are modulating greenhouse gases, providing clean water, maximizing carbon sequestration, promoting biodiversity and dispersing pests, pathogens and weeds. The redesign phase is likely to be the most transformative, presenting social, institutional, and agricultural challenges [37,38,39,40].

The food supply chain begins with the production of food in the agricultural sector, from which large amounts of waste or by-products are produced. These can be organic or derived from agri-food SWL, as is the case of low quality fruits and vegetables, damaged and/or unharvested products in the fields, by-products of low or zero commercial value, among others [41]. From an environmental point of view, the generation of agri-food SWL contributes to the upsurge of greenhouse gas emissions by its final discarding in landfills and during activities related with the production, processing, manufacturing, transportation, storage and distribution of food. Moreover, the generation of SWL also potentiates the lessening of natural resources in terms of soil, nutrients, water and energy, the disruption of biogenic cycles due to exhaustive agricultural activities and all other impacts typical of any stage of the food supply chain. At the economic level, the expenses related to food waste negatively affect the income of farmers and end users (consumers) and, socially, endorse greater food insecurity worldwide [42,43]. Hence, the reduction of food waste through the recovery of its valuable constituents presents an important approach towards increasing the overall sustainability of food systems, which gathers all the elements (environment, people, inputs, processes, infrastructures, institutions, etc.), the events linked to the production, processing, distribution, preparation, and consumption of food, and their socioeconomic and environmental outcomes [44,45]. 

## 4. Agri-Food Surplus, Waste and Loss

Every year, billions of tons of agri-food SWL are produced along the entire food supply chain, including all segments of waste management from collection to disposal [41,46]. The terms “food loss” and “waste” are usually used to identify materials intended for human consumption that are later discarded, degraded or contaminated [47]. However, the divergence between the different types of food waste and its appropriate categorization is of underlying importance so that they can be linked to a corresponding hierarchy, which represents a challenge for its prevention and for the maintenance of a sustainable system of management in the emerging circular economy. In recent years, the concepts applied in this hierarchy have not provided a clear and coherent scope of the different types of food waste, following in erroneous and broad estimates [48]. Different terms have been used to describe food waste, including “food loss”, “food waste” and “food surplus”. In defining these terms, five main stages in the food supply chain were considered, namely agricultural production, post-harvest activities, processing and manufacturing, retail and wholesale, consumption and services [47]. Although the term “food loss” generally refers to the post-harvest and processing stage, it can also include the loss of food suitable for human consumption but not marketable for aesthetic reasons [49,50]. Additionally, this term may as well be applied to unintentional losses of quantity or quality of the food products and, in this case, may overlap with that of food waste [51,52]. In turn, all edible leftovers produced, manufactured, retailed or served, suitable for human consumption, are part of the food surplus, thus referring not only to the retail and consumption phase of the food supply chain [53], but also to agricultural overproduction (e.g., primary phase) [43]. Ultimately, food waste includes foods that cannot be consumed by humans due to their natural inedibility or food handling along the entire food supply chain [48,54]. This category may include food originally produced for human consumption, but which has been discarded or not consumed, including food that is still edible and deliberately discarded.

The main reasons related to agri-food SWL during food processing and production activities are associated with damage caused through improper transport and storage of products, losses in processing or contamination, and improper packaging. Additionally, retail methods and markets also impact the generation of these food residues, mostly due to conservation or handling issues and lack of adequate storage facilities [41]. At the consumer level, the generation of food SWL is mainly due to food purchases above factual needs, excessive preparation of food amounts for consumption, poor storage conditions, and confusion between the terms “consume before” or “use until” [43]. Moreover, the production of these residues is influenced by the sociodemographic characteristics of the households, consumption behaviors and dietary patterns [55]. Although contradictory, the agri-food SWL generation is not limited to developed countries. Interestingly, reports from different countries around the world indicate that the generation of SWL has similar values in both industrialized and undeveloped countries, despite having different etiologies. In the latter, more than 40% of food losses occur in the post-harvest and processing stages, while in developed countries, the greatest losses occur at the retail and consumer level, with more food per capita being wasted in high-income countries [16].

According to Galanakis [56], waste arising from different segments of the food industry can be grouped into two main categories: plant origin (cereals, root and tubers, oil crops and pulses, fruit and vegetables) and animal origin (meat products, fish and seafood and dairy products). Depending on their source (except in the case of meat/fish), agri-food SWL may be less prone to spoilage when compared to waste produced at the end of the food supply chain (e.g., individual households), which tend to be widespread, making it challenging to recover their constituents due to the need for an additional step of collection and reduction of biological stability [56]. Specifically, agricultural residues from fruits and vegetables are the result of mechanical damage and/or spillages during harvesting and separation of post-harvest products to meet the quality standards of markets/consumers. According to FAO [16], about 42% of the fruits and vegetables produced worldwide are lost or wasted even before reaching the consumer, with a large sum being disposed in landfills or rivers, representing a threat to the environment due to their high biodegradability, leachate, and methane emissions [57]. These residues have a huge potential to be used for the recovery of value-added constituents, since they are a rich source of nutrients (minerals, phenolic compounds, sugars, proteins, fibers, and others) available for the production of new bio-based products [58,59,60]. Depending on the raw material, the waste generated during the processing of fruits and vegetables may contain peels, seeds, fruit, leaves, straw, stems, roots, or tubers [61]. Also, depending on the plant species and tissues, these residues may hold different properties, including flavoring and preservation (shells and seeds) [61], antioxidant and antidiabetic properties (tissues rich in carotenoids, vitamins and fibers), among others, capable of preventing certain diseases [62,63]. Thus, these compounds are of great relevance in human health and well-being [64], despite being often unstable when in contact with environmental and industrial process conditions, making it necessary to find a joint solution to the problems of waste management and resource depletion [65].

### The Particular Case of Carrots (Daucus carota L.), Lettuce (Lactuca sativa L.), and Broccoli (Brassica oleracea L. var. italica)

Carrots, lettuce and broccoli are some of the most commonly consumed and produced vegetables worldwide, mainly for their valuable nutritional and chemical characteristics, thus resulting in great amounts of SWL generated through the entire food supply chain, namely through cultivation, harvesting, storage, and processing, among others.

Carrots (*Daucus carota* L.) are one of the 10 most economically important and consumed vegetables worldwide. The species belongs to the Apiaceae family and has gained popularity for the nutritional composition of its edible taproots, positive health effects and characteristic aroma. The annual production of carrots worldwide is approximately 36 million tons, the main producer being China, followed by Russia, USA, Uzbekistan, Poland, Ukraine and the United Kingdom [66,67]. Nutritionally, carrot roots hold approximately 88% water, 7% carbohydrates, 3% fiber, 1% protein and 0.2% fat. Also, they are considered an excellent source of vitamins, biotin, minerals, phenolic compounds and carotenoids (β-carotene, α-carotene and lutein) [67,68,69].

Traditionally, carrots are used as a medicine for liver and kidney failure, skin diseases and burns, and hypotensive conditions [69]. Its beneficial effects on human health derive from its composition rich in compounds of interest. For example, their structure rich in carotenoids makes carrots an important food source in the prevention of diseases such as atherosclerosis, UV-induced erythema and cancer, while fibers play a protective role against coronary heart disease, diabetes, obesity and also some types of cancer. An adequate intake of minerals and phenolic compounds with different bioactive properties is also associated with the reduced risk of developing cardiovascular diseases and the modulation of several acute and chronic diseases [68,70,71,72].

To harness the potential of valorizing any food to its fullest extent, its nutritional composition must be known [73]. Thus, the range of values between which the energetic value, proximate and chemical composition, and the arrangement in macro and micro elements of *Daucus carota* L. are positioned are presented in Table 1. In the literature reports, the moisture content of carrots varies from 69.06 to 90.87% among different varieties. Carbohydrates were the most abundant macronutrient (up to 8.39 g/100 g fw), 7.18 to 8.87 g/100 g fw come from dietary fiber [74], followed by relatively high amounts of protein (6.46–10.73 g/100 g fw) and ash (1.12 to 7.37 g/100 g fw), with lipids being the least abundant (0.28 to 1.91 g/100 g fw) [68,74,75,76,77,78,79]. Glucose, fructose and sucrose were the major sugars identified and reported in carrots, with sucrose standing out as the most abundant one (2.663 g/100 g fw), followed by relatively low concentrations of glucose and fructose (1.137 and 1.153 g/100 g fw, respectively) [75,80,81,82]. Regarding organic acids, a study performed by Bonasia et al. [76] showed a total organic acids concentration of 333.5 mg/100 g fw in the variety “Carota a punta lunga”, with malic acid showing up as the most abundant in this variety (133.9 mg/100 g fw). Regarding mineral composition, Uzel et al. [83] report that potassium (K, 320 mg/100 g fw), sodium (Na, 69 mg/100 g fw), phosphorus (P, 35 mg/100 g fw) and calcium (C, 33 mg/100 g fw) are the four prevailing elements in black carrots, followed by magnesium (Mg, 12 mg/100 g fw), zinc (Zn, 0.24 mg/100 g fw), iron (Fe, 0.30 mg/100 g fw) and manganese (Mn, 0.143 mg/100 g fw).

Carotenoids are important secondary metabolites to which particular health related properties have been attributed. The carrot root, mainly the orange root variety, is one of the main sources of these pigments, which includes α- and β-carotenes and lutein. According to published data, the most abundant carotenoid found in *D. carota* is β-carotene, whose concentration may vary between 0.392 to 29.0 mg/100 g fw, followed by α-carotene (0.091 to 26.3 mg/100 g dw), and finally lutein (0.145 to 3.2 mg/100 g fw) (Table 2) [68,71,82,85,86,87]. Overall, phenolic compounds are important as they may hold antioxidant capacities, capable of increasing the oxidative stability of foods. Some of the phenolics identified in *D. carota* (Table 2) include 3-caffeoylquinic, 5-caffeoylquinic, feruloylquinic, and 5-feruloylquinic acids, as well as caffeic and di-caffeic acid derivatives. Specifically, *p*-hydroxy benzoic and salicylic acids together accounted for nearly 80% of the total phenolic acids in fresh carrots [4]. Similarly, lower amounts of gentisic; 2,4-dihydroxy benzoic; protocatechuic; *p*-coumaric; *o*-coumaric; vanillic; ferulic; syringic; chlorogenic; gallic and trans-cinnamic acids were acknowledged [88,89]. Ranjitha et al. [88] also reported the occurrence of total flavonoids (2.401 mg/100 g fw) and total phenolic acids (3.042 mg/100 g fw). Regarding its bioactive properties, *D. carota* showed good antioxidant [4] and anti-inflammatory activities, with purple carrots in particular inhibiting COX-1 and COX-2 in the range of 31 and 44%, respectively [78]. In addition, a total of 154.44 mg/g dw in anthocyanin compounds were quantified, with the acylated forms standing out as the most prevalent structure (83.0% of total anthocyanins) [78]. The analysis of phenolic compounds in purple carrots also allowed the identification of compounds belonging to the group of hydroxycinnamic acid derivatives (HCA, 133.72 mg/g dw), being present as esters, glycosides and glycoside-ester forms (Table 2) [78].

Large amounts of carrots are discarded every year because they do not meet market standards due to quality defects, with about 20–30% being thrown out due to irregular size, shape, or color [92]. Additionally, the carrot-processing industry (puree and juice) produces great amounts of waste and by-products, such as peels, that could be recovered and used as a source of valuable biochemical compounds [93]. Carrot pomace, for example, is the main by-product resulting from the extraction of carrot juice. During juice extraction, carrot pomace undergoes extreme mechanical stress. Moreover, plant tissues may respond to abiotic stresses, such as ultraviolet C (UVC) radiation, through the accumulation of bioactive compounds. In this sense, a study carried out by Sánchez-Rangel et al. [94] investigated the effects of UVC light on the accumulation of phenolic compounds and in the antioxidant activity in this residue. Their results showed that, in the untreated carrot pomace, there was an increase of 709.5% in total phenols and a good correlation of these with antioxidant activity. On the other hand, the residue treated with this type of radiation showed an increase of 143.6% in the concentration of chlorogenic acid after irradiation for 48 h, while the presence of protocatechuic and 3,5-dicapheoylquinic acids was confirmed; however, neither compound was detected in the control (untreated) group. This study allowed for the validation of the valorisation of carrot pomace through UVC radiation, enhancing its concentration in specific antioxidant compounds. Likewise, Chiboub et al. [95] investigated the chemical composition and antibacterial activity of essential oils extracted from the green tops (aerial parts) of *D. carota* (yellow and orange root varieties). The results showed the presence of large amounts of essential oils, mainly sesquiterpenes, which could inhibit Gram-negative bacteria. From this study, the potential of valorising essential oils from carrots by-products (green tops) was proven, which can be promoted as natural antimicrobials in food preservation systems, as well as the possibility of using these essential oils in the flavouring industry.

Broccoli (*Brassica oleracea* L. var. *italica*) is a vegetable that belongs to the Brassicaceae family, whose main edible parts are the shoots and immature inflorescences. The main producing countries of this vegetable are China, India, the USA, Spain, Italy, France and Mexico [96,97]. The edible portion of broccoli is characterized by its high water (89%) and low fat content (0.37%), as well as lower amounts of proteins, dietary fibers and carbohydrates. Broccoli is also a valuable source of minerals such as potassium, phosphorus, calcium and sodium, vitamins (especially vitamin C, A and folic acid), glucosinolates, polyphenols (flavonoids and hydroxycinnamic acids), among others [96,97,98,99,100]. Therefore, eating habits that include broccoli in the regular diet are important in the prevention of chronic diseases, such as cardiovascular diseases and cancer, as they may perform antioxidant, antimicrobial, and other bioactivities that prevent oxidative stress related to different conditions [96,101,102,103]. The nutritional assets of broccoli, as well as its fatty acids profile and micro/macro elements composition are presented in Table 3. The analysed reports showed that broccoli may hold a moisture content ranging from 6.93 to 9.59 g/100 g dw [104]. In different varieties and/or cultivars, the protein content of broccoli may show great variation with values between 4.39 to 28.99 g/100 g dw [101,105,106,107,108], the same happening with fat and ash contents (4.38 to 10.01 g/100 g dw and 6.85 to 15.74 g/100 g dw, respectively) [101,104,105,108,109]. Moreover, according to Shi et al. [108] this vegetable has about 55.7 g/100 g dw of carbohydrates, of which 8.85 to 55.34 g/100 g dw may be attributed to dietary fiber [105,107,108,109]. The fatty acids composition is quite diverse, with erucic acid (C22:1n9) appearing as the major compound (32.40–48.00 g/100 g dw), and stearic acid (C18:0, 1.3 g/100 g dw) as the less abundant one [105]. According to several authors, the most abundant mineral in broccoli is potassium, (K, 13.04 to 182.0 mg/g dw) and the least abundant is selenium (Se, 0.00016 to 0.00023 mg/g dw) and copper (Cu, 0.00021 to 0.00029 mg/g dw) [105,110]. The variability of results in all parameters is mainly due to differences between samples, namely country of origin, growing conditions, and different cultivars and/or varieties analysed.

The bioactive compounds, glucosinolates composition, as well as the antioxidant and antitumor activities of broccoli are described in Table 4. Regarding carotenoids, only β-carotene and lutein are described by various authors [111], in a concentration of 9.06 mg/100 g dw and 0.6795 mg/100 g dw, respectively. Over the years, different phenolic compounds have been described in broccoli. However, the phenolic compound with the highest concentration described in the reported data is 3-caffeoylquinic acid (2.5 to 11.51 mg/100 g dw), whereas Q-3,7-*O*-digluc (0.011 mg/100 g dw) was detected in the lowest concentration [100,112]. According to Thomas et al. [100], the total polyphenols content may vary between 7.45 to 25.04 mg/100 g dw. As for the content of proanthocyanidins and total ascorbic acid, a concentration of 125 and 95 mg/100 g dw, respectively, was identified [107]. Chemically, broccoli contains various glucosinolates, and several authors have reported the composition in these compounds in different varieties and/or cultivars. Here, the major compound reported is glucoraphanin (0.083–6.004 mg/100 g dw) [101,110,113,114,115,116], followed by 3-methylsulfinylpropyl and 4-methylsulfinylbutyl. Regarding the content of total aliphatic and total indolic glucosinolates, the authors reported values ranging from 0.08 to 1.52 mg/100 g dw and 0.17 to 6.54 mg/100 g dw, respectively [113,114,116]. The content of total glucosinolates is reported by several authors with values from 0.75 to 9.12 mg/100 g dw [111,113,114,116,117]. The antioxidant activity of broccoli has also been evaluated using the DPPH, FRSA, total phenolic, total polyphenols, and total flavanols assays, with reported results showing its bioactive potential [100,102,107,118,119]. Additionally, Bachiega et al. [120] evaluated the antitumor activity of *B. oleracea* var. *italica*, against U251, MCF-7, 786-0, NCI-H460, HT29, and the HaCaT cell lines, which presented IC_50_ values indicative of a good performance in these fields.

Throughout the broccoli supply chain, there are many losses of plant materials generated during agricultural production, processing, distribution and consumption [119]. Broccoli flowers typically represent 10 to 15% of the total plant biomass that are consumed or used in large-scale preparations of pre-cut and frozen vegetables. The residues are in the form of leaves and stalks that are usually discarded, and flowers that are too mature or with some yellow spots. The highest percentage of residues occurs at the post-harvest stage due to the high quality standards established, with losses in the range of 45 to 50% of total harvested broccoli [120,121]. Moreover, around 20 to 25% extra losses happen in the field, producing large amounts of florets, stems, and leaves as crop residues [121]. Landin-Sandoval et al. [122] showed the potential of using broccoli stalks, resulting from the processing of the food industry, as adsorbents for heavy metals and/or their transformation into carbon-based materials to control environmental pollution. These were prepared from pyrolysis and carbonization processes and showed to be promising adsorbents in the removal of metal cations/heavy metals from aqueous solutions. Other studies highlighted the use of different agri-food SWL from broccoli as antimicrobial agents [123], phytonutrients [124], and in the extraction of bioactive compounds for incorporation into food products [125].

Lettuce (*Lactuca sativa* L.) belongs to the botanical family Asteraceae, being one of the most consumed salad vegetables worldwide. Its main producers are China, USA, India, Spain, Iran, Italy and Japan [126,127,128]. Lettuce is mainly consumed for its whole tender leaves or as a minimally processed product [129], being known for its high content in macronutrients (K, Na, Ca and Mg) and trace elements (Fe, Mn, Cu, Zn and Se) that are essential for human’s health and nutrition. This vegetable is also known as a good source of photosynthetic pigments (chlorophylls and carotenoids), vitamins (B, A, C and K) and phenolic compounds that benefit nutrition and play a significant role in preventing various diseases [126,127,129,130]. Its polyphenolic profile is mainly composed of hydroxycinnamic acids, represented by derivatives of caffeic acid and flavonols, rarely found in its free form (glycosylated derivatives of quercetin and kaempferol) [129,131]. From traditional knowledge, lettuce is often used in the treatment of a variety of disorders, such as insomnia, dry cough, rheumatic pain, anxiety, inflammation and stomach problems, being also recognized for its antioxidant, neuroprotective, antiproliferative and antitumor properties [129,132,133,134].

According to the reported data (Table 5), the moisture content of different varieties of *L. sativa* may range from 91.6 to 96.1 g/100 g of fw [135,136], as well as proteins (0.004 to 1.90 g/100 g fw) [135,136,137,138,139,140,141,142], and fat (0.20 to 0.49 g/100 g fw) [135,136,138]. Ramos-Sotelo et al. [135] found that the ash content in *L. sativa* var. tropicana M1 was 0.88g/100 g fw. In turn, the carbohydrate, energy and dietary fiber content was reported to be 0.83 g/100 g fw, 11.5 Kcal/100 g fw and 1.18 g/100 g fw, respectively [136]. Again, the variability of the results in all parameters is due to differences in the country of origin and year in which the samples were produced, growing conditions, and the different cultivars and/or varieties in each study. The fatty acids composition of lettuce was reported by Kim et al. [143], who studied three different varieties, *L. sativa* L. var. capitata, *L. sativa* L. var. crispa and *L. sativa* L. var. longifólia. According to this study *L. sativa* L. var. capitata was the one with the highest levels of palmitic (C16:0, 15.71%) and α-linolenic acids (C18:3n3, 61.77%), with *L. sativa* L. var. crispa presenting the highest amount of linoleic acid (C18:2n6c, 15.69%). Regardless of the studied variety, lettuce may present a quite diverse mineral composition, with zinc (Zn, 0.047 to 27.4 mg/100 g fw) appearing as the most prevalent mineral in this species, followed by phosphorous and magnesium [129,143,144,145,146,147,148,149].

The content in carotenoids, vitamins, phenolic and bioactive compounds of *L. sativa* L. are described in Table 6. Regarding carotenoids, the authors reported the presence of β-carotene in this species in the range of 0.51 to 30.61 mg/100 g fw [144,149]. Also, its composition in vitamins, especially vitamins A, B and C was also described. According to Yoshida et al. [136], the concentration in vitamin A in this vegetable was 59 mg/100 g fw, with vitamin B1 appearing with the lowest concentrations (0.03 mg/100 g fw). As for the phenolic compounds described in lettuce, the most abundant compound identified was isorhamnetin (1.77 to 6.17 mg/100 g fw) [129], followed by significant amounts of quercetin (0.04-5.23 mg/100 g fw). Also, the total flavonoid content of *L. sativa* ranges from 1.44 to 6.0 mg/100 g fw, total phenolic acids from 0.001 to 18.70 mg/100 g fw, and finally total phenols from 0.001 to 25.50 mg/100 g fw [129,142,144,149,150,151,152]. Moreover, the anthocyanin content was reported to vary between 0.001 to 16.0 mg/100 g fw, depending on the varieties under investigation [141,142,144,149]. The antioxidant activity has been evaluated using different assays, namely DPPH, ABTS, FRAP and AA. According to several authors, the DPPH method comprised values between 0.003 and 54,760 mg/100 g fw, the ABTS method values between 0.005 and 6.05 mg/100 g fw, FRAP with values ranging from 15.590 to 127.57 mg/100 g fw and AA with 22.30 a 96.90 mg/100 g fw [129,134,139,143,144,145].

Annually, about 93% of lettuces are intended for fresh use, and only 7% are intended for processing purposes [154]. Lettuce residues are comparatively low, because when they do not meet specifications or are unsuitable for fresh markets, the crops are mainly used in the processing industry. According to research data, only 5% or less of lettuce is directly wasted, and the core and outer leaves represent about 10% of the waste that is used as green manure or animal feed [154]. These cannot be used for other purposes due to their high water content and low nutritional value. On the other hand, the packaging produces large amounts of waste, namely leaves and stems, among others, which can reach about 50% of the material harvested during lettuce production, and which are extremely perishable. Llorach et al. [155] showed that the by-products of three different varieties of lettuce are excellent carriers of phenolic compounds with antioxidant capacity, and therefore these residues can be used, from an industrial point of view, as a rich and cheap source of antioxidant compounds capable of functionalizing foods.

## 5. Efficient Management and Valorization of Agri-Food SWL

The sustainable management and valorization of food SWL worldwide is mainly triggered by environmental legislation, the need for sustainable use of natural resources through technological development, and by the high costs of waste disposal and management. Yet, disposing of agri-food SWL is often a challenge, as these residues can be difficult to manage. Here, different sorts of food waste can be altered by the microbiological activity of the microorganisms they have, becoming biologically unstable and propitious to the development of pathogens. If these wastes are not properly processed, unacceptable hygienic conditions may result, with the growth of larvae, microorganisms and fungi, which further generate strong odors. Similarly, fat-rich agri-food SWL are more prone to oxidation, leading to the release of foul-smelling fatty acids, and the acceleration of decomposition due to continuous enzymatic activity [156]. The management of agri-food SWL may involve several methods of physical, chemical, thermal and biological treatments, with the main strategies to promote its minimization and valorization being incineration, anaerobic fermentation, composting, landfill, or its use as animal feed or fertilizers [157]. Some reports about potential applications of agri-food SWL in the development of new value-added food products are presented in Table 7.

The utilization of food SWL as animal feed is one of the most common conventional practices, as these residues are rich in fat and proteins, suitable for feeding omnivorous animals [158]. Specifically, the valorization of fruits and vegetables as animal feed has been considered a traditional practice worldwide [158]. As an example, the utilization of vegetable waste as feed on lactating cows improved the amount of α-linolenic acid and cis-9, trans-11 CLA in milk [159]. However, the potential presence of toxic compounds with anti-nutritive effects and an unbalanced nutrient composition must be considered, as it may lead to harmful effects for both humans and animals [157]. Hence, efficient pre-treatments should be performed to ensure quality, feasibility, and low cost for a potential scale-up of this practice [160].

On the other hand, food SWL resulting from food processing may hold large amounts of organic substances that can be converted into energy which, ultimately, can be recovered in the form of heat or electricity. Particularly, fruit and vegetable residues are considered an underexplored resource with high potential for energy production [161]. Here, the main biofuel conversion methods are anaerobic digestion and thermochemical treatments, employed according to the moisture content of the used residues [157]. For example, through incineration, heat was produced over the oxidation of combustible material from agri-food SWL. However, this results in high emissions of gases into the atmosphere, causing negative and costly environmental impacts [157]. In anaerobic digestion, a wide range of microorganisms are used to stabilize food residues in the absence of oxygen. This technique has gained special attention as an effective pretreatment in the valorization of fruit and vegetable wastes under controlled conditions [162].

**Table 7:** Potential applications of agri-food SWL in the development of new value-added food products.

Agri-Food SWL	Application	Added-Value on Functional Foods	Reference
Seed, pomace, and grape peal	Baked products and pasta	Boost of functional ingredients without quality depletion of products	[163]
Watermelon seeds	Biscuits	Quality and protein content improvement	[164]
Passion fruit pulp and pomace	Fermented and non-fermented beverages	Source of probiotic food carriers; increased shelf-life of the final product	[165]
Pigeon pea cotyledons	Biscuits	Protein and fiber content enhancement	[166]
Grape seeds	Cereals, pancakes and noodles	Improved antioxidant activity	[167]
Sugar beet pulp	Foods, beverages	Flavoring agent	[168]
Grape peel, seeds, and remains of the pulp	Biscuits	Flour with physicochemical characteristics within the nutritional standards	[169]
Pineapple peels	Cereal bars	Increased fiber content and	[170]
Plum	Foods and beverages	High concentration in polyphenols and flavorant agent	[171]
Grape pomace	Biscuits	Increased protein, fiber, and ash content	[172]
Apple pomace	Sorghum and corn extrudates	Improved phenolic content, antioxidant activity, textural, and functional properties	[173]
Pomegranate, grape, and rosehip seeds	Turkish noodles	Increased antioxidant activity by 5.7 to 8.4 times	[174]
Tomato skin	Foods and beverages	Reduces browning and increases shelf-life up to 9 days; presence of bioactive compounds	[175]
Olive pomace	Oat and rice extrudates	Advantageous effect on the physical characteristics; high content of fiber, protein, and polyphenols	[176]
Apple pomace and sugarcane bagasse	Corn extrudates (high fiber croquettes)	Extrudates with considerable expansion, with comparatively lower energy contributions and high fiber content	[177]
Carrot pomace	Cookies	Increased total carotenoids content and total dietary fiber	[178]
Artichoke and broccoli	Cheese	Improved total phenolic and total flavonoids content	[179]
Pumpkin pomace	Bread	Total carotenoids improvement	[180]

Here, the degradation of organic substrates occurs, and the residual slurry can be used in the biopolymer industry and as fertilizer [181], while biogas is produced and used to generate electricity through thermal energy [182]. In addition, a study performed by Bres et al. [183] showed that the co-digestion of poultry manure using plant residues in a semi-continuous manner resulted in the production of 31% more biogas and methane compared to the individual digestion of the former. However, it must be taken into account that an appropriate control and optimization of the treatment of fruit and vegetable wastes must be provided, namely variations in pH, temperature, alkalinity, moisture content, and dosage of microorganisms to obtain the desired efficiency [184].

Composting, in turn, results from the aerobic degradation of organic materials into relatively stable products, by the action of fungi, bacteria and protozoa [185] to produce manure or fertilizers. Additionally, a biomass capable of improving soil structural properties, water and nutrient capacity, the maintenance of living soil organisms and the return of organic materials into the soil is also generated [186]. Reactions derived from the catabolism of hydrolysis and oxidation of the carbonic substrate produce CO_2_ and heat, which enhance microbial growth and suppress their metabolic needs [187]. This process is affected by physicochemical parameters such as pH, humidity, C/N ratio, temperature, aeration and partial size [188]. Over the years, different composting methods have been developed, making this a viable technology for the treatment of fruit and vegetable waste. Here, one of the most used techniques is vermicomposting, which uses different species of earthworms to convert organic matter into compost [189]. This can be efficiently used to sequester CO_2_ as soil carbon, thus reducing greenhouse gas emissions from the soil into the agricultural ecosystem.

Various commonly used enzymes, such as amylase and cellulase, have been gaining attention given its ability to break down into β-glucose, and its high efficiency in biofuel production [190]. These biofuels can be an efficient option as a source of petroleum fuel while lowering greenhouse gas emissions and causing less damage to the environment. For example, the efficient application of cellulase has been conducted to generate bioethanol using sugarcane bagasse [191]. Similar studies using a mixture of fruit wastes and *Saccharomyces cerevisiae* post-acid hydrolysis [192], showed that this pre-treatment might improve conversion of waste into a fermented sugar, such as the aid of the enzyme in producing ethanol.

One of the cheapest techniques for disposing of food waste is in water courses, which represents a major threat not only to marine life but also, indirectly, to human life. In this field, adsorption is considered an efficient technology in the treatment of effluents. However, traditionally, this technique uses activated carbon, clay or silica for this purpose, causing great increasing its cost. Therefore, fruit and vegetable residues, which have a high porosity, have been studied as an effective alternative in the implementation of this technique without resorting to the aforementioned compounds [61,193]. Fibers from fruit and vegetable residues have also been used to remove heavy metals, pesticides or pollutants, a capacity that comes from their constitution in functional compounds, such as aldehydes, alcohols and ketones, which are associated with the surface of the adsorbate, removing contaminants [194].

Given the exponential increase in CO_2_ emissions during fuel combustion, techniques for compacting and binding bulky food waste as a fuel resource have been studied by several researchers over the years. In this field, and given the current assumptions for valuing food waste, these have been used as a resource for briquetting [195]. Among the common types of briquettes, mixtures of agro-waste and coal-agri-waste have gained prominence due to their high thermal efficiency. Since plant residues ignite at lower temperatures compared to coal, less smoke and more heat are released [196]. In addition, the lignin present in the residue provides high calorific value and assists in the binding of particles leading to the formation of briquettes or pellets. Despite the good results resulting from the valorization of food waste in the production of briquettes as an energy resource, techno-economic factors, the availability of necessary material and marketing strategies must be taken into account [184].

Compared to conventional methods of managing food waste, in which large volumes are required for the production of value-added products, emerging techniques aim at the production of these same compounds starting from green extraction processes and with the need for smaller volumes of waste [184]. The current demand for food supplements has become an increasing reality with the rise of health problems related to food. In this field, fruits and vegetables emerge as important products that contain essential compounds to promote health. A nutraceutical can be defined as a compound derived from a food or part of it capable of providing benefits to human health, classified as antioxidant, dietary fiber, fatty acids, or polyphenols, among others [161], to which different promising bioactivities in the nutraceutical industry are attributed [193,197,198,199]. These bioactive ingredients can be extracted from different agri-food SWL, such as fruits and vegetables, and integrated into different food products, thus providing biodynamic attributes. 

Over several decades, plastic has been an integral part of various human activities, given its lightweight nature, low-cost, and high durability compared to other materials. However, its non-biodegradable nature has negatively affected all ecosystems [200], driving the shift towards more ecologically acceptable materials or biocomposites made by different mixtures of agri-food SWL. In this field, food residues from fruits and vegetables, as potential films for the production of biopackaging, have been explored as an alternative to plastic. By presenting good mechanical properties and environmental benefits at low cost, cellulose nanocrystals and chitosan extracted from different plant residues (e.g., mango residues) and incorporated with polyvinyl alcohol proved to be efficient as an active film in edible packaging [201]. Also, cassava peel starch and chitosan (shrimp waste) seem to provide the correct packaging of smoked products [202]. Thus, the production of bioplastic from agri-food SWL, mainly vegetables and fruits, can be considered a sustainable technique in the management and recovery of food waste due to its biodegradability and carbon neutrality [203].

The exploration of agri-food SWL from fruits and vegetables as possible flavoring agents is still a little explored area. Flavor is an essential characteristic in the acceptance of food, and those of natural origin present in various plants and flowers can be applied in different sectors (cosmetics, perfumes, surfactants and the food industry as additives), to the detriment of artificial ones [204]. Fruit and vegetable residues are excellent candidates for flavoring other foods, as is the pineapple canned waste, which is a source of ferulic acid and aromatic compounds, such as vanillic acid and vanillin [205]. The yeast *Saccharomyces cerevisiae* is also used in the fermentation of orange peel residues, resulting in the production of flavoring compounds (α-terpineol and limonene) [206]. Despite the promising results in the isolation of different ingredients, their volatility and sensitivity to environmental conditions must be explored.

Nanoparticles have been effectively explored in different medicines, electrocatalysis, sensing, and drug delivery, given their small dimensions and larger surface area that give them high reactivity and porosity, as well as cellular barriers. Its synthesis can be carried out through different physico-chemical methods. However, these have a high cost and negative environmental and safety effects [207]. Therefore, in recent years, different agri-food SWL have been used in the green synthesis of nanoparticles [208]. For example, gold nanoparticles have been studied for their biomedical, cytotoxicity and biocompatibility applications [209]. The antibacterial properties of gold nanoparticles from the leaves of *Pergularia daemia* have been proven, presenting resistance to bacteria such as *Escherichia coli*, *Pseudomonas aeruginosa*, and *Bacillus subtilis* [210]. Similar effects were seen in the use of mint leaves in cotton fabric, where the synthesized gold nanoparticles provided UV protection and antibacterial sensitivity against food pathogens (*E. coli* and *Staphylococcus epidermidis*). Thus, the production of gold nanoparticles may bridge large gaps in medicine regarding the bacteria that colonize and damage medical devices.

Despite the relative effectiveness of applying these agri-food SWL minimization and valorization approaches, the “Community Strategy for Waste Management” stated that waste prevention is the best option for its minimization, followed by reuse, recycling and energy recovery. Furthermore, according to this directive, landfill disposal and incineration with low energy rescue are considered the worst strategies from an environmental point of view.

## 6. Recovery of Agri-Food SWL

Until very recently, the potential of food waste to generate new opportunities and markets was misjudged. Nevertheless, consumers’ perception about environmental issues and legislation has increased the demand for new methodologies for waste recovery instead of disposal. Thus, over the last few years, new methods have been developed and implemented, aiming at recovering and reusing valuable components of these types of residues, as conventional methods only contribute to their partial utilization [159].

**Figure 2:**
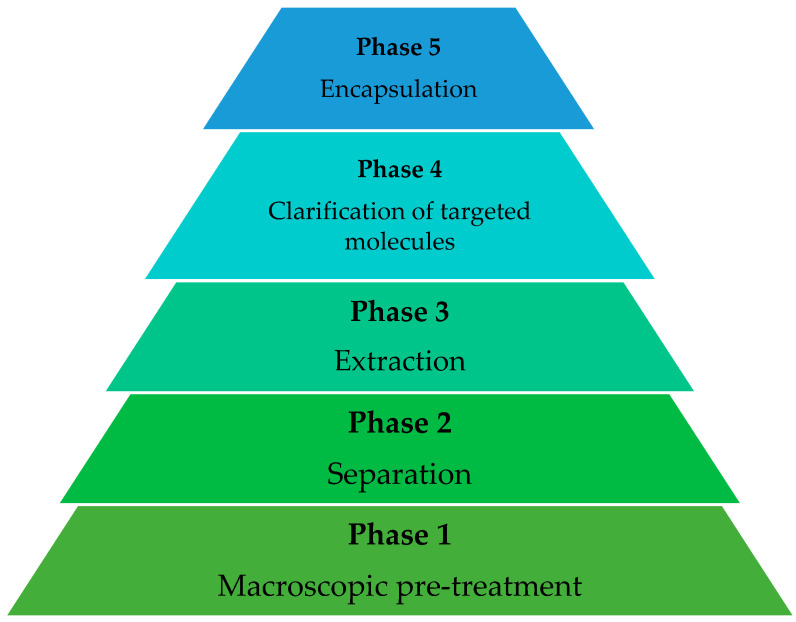
Stages of recovery of compounds of interest from agri-food SWL.

The recovery of compounds of interest from agri-food SWL and by-products is regulated by advanced analytical chemistry standards, which comprise the stages of macroscopic pre-treatment, macro- and micromolecules separation, extraction, isolation and preparation, and product formation (Figure 2). Depending on the matrix, one or two steps can be withdrawn and/or change order [56].

In the macroscopic pre-treatment phase of agri-food SWL and/or by-products, the water, solids and fat content is adjusted, enzymes are activated or deactivated, the microbial load is managed, and an increase in permeability is promoted. Depending on the nature and structure of the substrate, that is, whether it is solid, sludge or wastewater, various processes may be implicated. Thermal concentration is applied, for example, to fruit and/or vegetable pulps and apple pomace, while the thermal dehydration, through mechanical pressing or lyophilization, is only used to avoid the loss of thermolabile compounds and functionality. The disadvantage of this in relation to the former is related not only to the increase in cost but also to the lack of microbial pasteurization which, consequently, ends in a low shelf-life of the matrix being treated. Therefore, new techniques, such as foam mat drying, have been implemented to remove water from viscous and thermosensitive matrices (e.g., mango stern or apple puree) [211], given their lower requirements in terms of temperature and drying time [212]. Other techniques, such as centrifugation and microfiltration can be applied at this stage, given their competence to remove solids, oils and fats, which can inhibit mechanical managing and cause substrate degradation by auto-oxidation [213,214]. Regarding by-products derived from fruits and vegetables, wet grinding can be accomplished to improve the diffusion of extractants within the matrix. In the case of the recovery of compounds with particular characteristics (retake of phytosterols), an additional step of hydrolysis at high pressure and temperature, or saponification with alkaline solution is required [215].

Regarding the second stage of recovery of agri-food SWL and by-products, the most important concern is the effective separation of the matrix compounds from the agri-food residues, which can be accomplished by separating them from macroscopic to macromolecular and finally to micromolar size [56]. Small compounds (e.g., antioxidants, acids and others) are detached from macromolecules, such as proteins or dietary fibers, through the precipitation of the insoluble residue in alcohol. However, this technique is not capable of separating the complexes between smaller and larger molecules. Thus, more efficient approaches, such as membranes and ultrafiltration techniques [216,217], are required. Likewise, the isoelectric precipitation allows the selective precipitation of proteins, altering the pH into their isoelectric point [218]. These can also be separated by sonocrystallization, which promotes faster and more uniform crystalline growth compared to other conventional methods. This technique has been used to speed up the removal of whey protein during lactose recovery [219,220]. More recently, the implementation of colloidal gas aphrons with cationic and non-cationic surfactants has been investigated, with a view to selective separation of reverse-charged macro and micromolecules from residual liquids. On the other hand, pressurized microwave-assisted extraction has been indicated for accelerating the recovery of metabolites such as terpenes, flavonoids or pectins from orange peels, although this technique is difficult to control and causes the degradation of thermolabile ingredients [221].

The extraction phase is the most relevant in the food waste recovery process, aiming at the solubilization of free molecules and the dissociation and subsequent solubilization of bound compounds [221]. Generally, different solvents are used in the separation of target compounds from the pre-treated residual matrix. Pressurized processes are used to improve the extraction of compounds (e.g., phenols and carotenoids), while distillation is used to recover aromatic molecules. Given its effectiveness, easy handling and moderate amounts of solvent required, the ultrasound-assisted extraction has captured the interest of researchers in the last decade. This, when applied in association with other techniques, such as steam diffusion or hydrodistillation, increases the ability to extract volatile compounds or essential oils from citrus products [222]. Additionally, through this technique, phenols can be extracted from grape seeds, betalains from red beetroot and pectin from apple pomace [56,223,224]. The supercritical fluid extraction, in turn, is an emerging technique applied to more arduous separation processes of compounds present in low concentrations in food waste, requiring low amounts of solvent for this purpose [225]. Also, the photodynamic effect induced by laser irradiation to food residues has been shown to promote minimal heating and enhance the extraction of aromas, anthocyanins, polysaccharides and proteins. This methodology does not require the use of solvents and is automated. However, it appears to be too sophisticated a technique to be implemented in this extraction phase [226].

The fourth stage of recovery includes the clarification of target molecules from the concomitantly extracted impurities, in which the adsorption process is used. Through this, specific low molecular weight compounds, such as antioxidants, are isolated from dilution solutions with a high capacity for this effect, as well as insensitivity to toxic substances [227]. This, however, is a time-consuming process, which uses high amounts of solvents, and requires a greater study of the sorption behavior of each component individually [228,229,230]. The aqueous two-phase separation, in turn, has been shown to be effective in the isolation of proteins and enzymes from crude cell extracts, and has recently been used in the fractionation of β-lactoglobulin and α-lactalbumin from whey [231]. This is an appropriate technique for the isolation of labile compounds, despite having long separation times and several processing steps. Furthermore, membrane processes are able to perform direct separations between compounds of different nature through reverse osmosis and nanofiltration techniques; this technique has been used to purify lactic acid in the recovery of whey proteins. Also, Bhattacharjee et al. [232] were able to purify whey proteins up to 90% using two-stage ultrafiltration coupled with ion-exchange chromatography, a very selective technique but much slower compared to the previous one.

The last recovery phase must necessarily involve the encapsulation or drying process, since extracts, residues and enriched elutions cannot be released on the market without guaranteeing the preservation of the properties of the compounds of interest. Through encapsulation, valuable compounds are trapped inside a coating material that guarantees their stability and masks unwanted organoleptic characteristics, protecting them against different environmental stresses and non-functional interactions with food matrices. Some polysaccharides (e.g., starch, cellulose, inulin, pectin, etc.) and proteins are commonly used as coating materials in these types of processes. When the compounds of interest are macromolecules, encapsulation is switched by a direct drying process such as spray drying, a well-known technique in the food industry for being easy to handle, continuous and economical [221]. However, it promotes the thermal destruction of labile antioxidants and low molecular weight volatile phenols. Recently, carotenoids (lycopene, α-carotene and β-carotene) and phenolic compounds (e.g., quercetin, caffeic acid and coumaric acid) from tomato residues and wine lees have been effectively encapsulated [56]. Lyophilization, on the other hand, is a more tenuous process that allows the preservation of labile antioxidants, spending more time and energy compared to the previous technique. In turn, melt extrusion is used to increase the palatability of polysaccharides, such as starch, and encapsulate flavors or nutrients. This requires less consumption of chemicals and water, and generally results in low yields. Liposomes and emulsions are also widely used to entrap compounds of a lipophilic nature or hydrophilic antioxidants (potato peel phenols) prior to their inclusion in rapeseed oil blends [232]. More advanced techniques perform encapsulation through the use of nanoemulsions, which are quite stable and provide a monitored delivery triggered by moisture and pH [233]. Reports have shown that nanoemulsions improve the ability to diffuse fat-soluble β-carotene in water and boost its intestinal bioavailability [234]. Furthermore, the formulations obtained can be used as natural coloring in water-based foods or as a mask to hide the taste and odor of tuna oil [235].

## 7. Conclusions

The rapid depletion of natural resources, environmental pollution and global warming have encouraged countries around the world to implement circular economy principles, given that humanity has always had linear economic systems characterized by the exacerbated consumption of resources beyond the capabilities of planet Earth. The agri-food value chain can be widely changed to achieve more sustainable agriculture practices, with natural resources recovery being one of the most relevant aspects. In this field, the valorization of agri-food SWL and by-products through its recovery and reuse is a challenge all over the world and, mainly, in developing countries, where techniques to add value to these wastes using only available resources may be explored. Currently, several food companies are implementing agri-food SWL biomass sustainable recycling systems, producing food, medicines, biologically active compounds, biomaterials and promoting sustainable energy generation. The agri-food residues are valuable sources of compounds of interest, such as proteins, phenolic compounds, and vitamins, among others, known for their beneficial effects in human health and well-being. Fruits and vegetables are one of the main sources of nutrients and bioactive compounds, from which huge amounts of waste are generated every year. Therefore, different strategies for reuse and development of new products have been developed, promoting their reuse in the food, pharmaceutical and environmental sectors. Lettuce, carrots and broccoli are among the most consumed vegetables in the world, from which large amounts of waste are generated along the food supply chain. However, carotenoids, phenolic compounds, and dietary fiber, among others, can be recovered from their residues and by-products, aiming at their reintroduction into food industry and in other sectors.

Thus, this review summarizes the foundations of sustainable agriculture practices through the efficient management of agri-food SWL, empathizing in the available conventional and emerging techniques to recover these type of residues, which is of vital significance in the maintenance of ecosystems. Moreover, we provide a full nutritional and chemical assessment of three worldwide consumed vegetables, namely carrots, broccoli, and lettuce, and the potential utilization of their SWL in different processes and formulations. Therefore, more studies that allow the sustainable recovery and reuse techniques of various types of food waste through different techniques should be conducted, with the ultimate goal of protecting our planet from the irrational use of natural resources exercised by man.

## Figures and Tables

**Table 1:** Energetic value, proximate and chemical composition, and micro and macro elements of *Daucus carota* L. taproots.

Proximate Composition (g/100 g fw)		References
**Moisture (%)**	69.06–90.87	[74,77]
**Energy (kJ/kg)**	552.7	[77]
**Carbohydrates**	0.6–8.39	[74,76]
**Protein**	6.46–10.73	[74]
**Crude fat**	0.28–1.91	[68,77]
**Ash**	1.12–7.37	[68,75,77,79]
**Dietary fiber**	7.18–8.87	[74]
**Sugars free (g/100 g fw)**		
**Glucose**	0.02–1.7	[75,76,81,82]
**Fructose**	0.05–1.5	
**Sucrose**	0.5–3.3	
**Organic acids (mg/100 g fw)**		
**Quinic Acid**	13.3–64.7	[76]
**Malic Acid**	26.0–266.2	
**Ascorbic Acid**	2.6–11.1	
**Oxalic Acid**	5.2–5.4	
**Macro and micro elements (mg/100 g fw)**		
**Ca**	29–37	[83,84]
**Mg**	6–12	
**Zn**	0.24–1.0	
**Na**	69	[83]
**K**	320	
**Fe**	0.30	
**Mn**	0.143	
**P**	35	

Ca—Calcium; Mg—Magnesium; Zn—Zinc; Na—Sodium; K—Potassium; Fe—Iron; Mn—Manganese; P—Phosphorus. For uniformity reasons, some values were converted from a dry weight basis or other units presented in the original source to a fresh weight basis.

**Table 2:** Carotenoids, phenolic compounds, antioxidant and anti-inflammatory activities of *Daucus carota* L. taproots.

Carotenoids (mg/100 g fw)		References
**α-carotene**	0.091–26.3	[68,71,82,85,86,87]
**β-carotene**	0.392–29.0	
**Lutein**	0.145–3.2	[68,71,86,87]
**Total carotenoids**	0.628–58.5	[68,81,82,85,86,87,88,90,91]
**Phenolic compounds (mg/100 g fw)**		
**3-caffeoylquinic acid**	4560	[67]
**5-caffeoylquinic acid**	579	
**feruloylquinic acid**	670	
**5-feruloylquinic acid**	89	
**caffeic acid derivative**	216	
**di-caffeic acid derivative**	3977	
***p*-Hydroxy benzoic acid**	1.143	[88]
**Salicylic acid**	1.212	
**Ferulic acid**	0.112–0.475	[88,89]
**Syringic acid**	0.21–0.807	
**Chlorogenic acid**	0.008–4.658	
Anthocyanins (mg/g dw) *		
**Cy 3-xyl-glc-gal**	8.77	[78]
**Cy 3-xyl-gal**	17.43	
**Cy 3-xyl-sin-glc-gal**	8.63	
**Cy 3-xyl-fer-glc-gal**	97.62	
**Cy 3-xyl-p-coum-glc-gal**	19.63	
**Pg 3-xyl-fer-glc-gal**	1.98	
**Pn 3-xyl-fer-glc-gal**	0.37	
**Total Anthocyanins**	154.44	
HCAs derivatives (mg/g dw) *		
**5-CQA**	80.17	[78]
**4-CQA**	19.86	
**CAD**	12.64	
**Total HCAs derivatives**	133.72	
**Antioxidant activity (mg/100 g fw)**		
**FRAP**	10.40	[88]
**Anti-inflammatory activity (%)**		
**Anti-inflammatory COX-1**	31	[78]
**Anti-inflammatory COX-2**	44	

* Could not convert results to fw; HCAs—Hydroxycinnamic acids derivatives; FRAP—Fluorescence recovery after photobleaching; COX—Cyclooxygenase-1 or 2. For uniformity reasons and when possible, values were converted from a dry weight basis or other units presented in the original source to a fresh weight basis.

**Table 3:** Energetic value, proximate composition, fatty acids, and micro and macro elements of *Brassica oleracea* L. var. *italica*.

Proximal Composition (g/100 g dw)		References
**Energy** **(kcal/100g dw)**	341.68–347.85	[105]
**Moisture**	6.93–9.59	[104]
**Protein**	4.39–28.99	[101,105,106,107,108]
**Crude fat**	4.38–10.01	[104,105,108,109]
**Ash**	6.85–15.74	[101,105,108,109]
**Carbohydrates**	55.7	[108]
**Fiber**	8.85–55.34	[105,107,108,109]
**Main fatty acids (%)**		
**C16:0**	4.30–6.90	[105]
**C18:2n6c**	12.20–13.00	
**C18:3n3**	1.50–2.20	
**C18:1n9c**	8.90–10.50	
**C18:0**	1.3	
**C20:1**	13.10–22.80	
**C22:1n9**	32.40–48.00	
**Macro and micro elements (mg/g dw)**		
**P**	0.01–8.17	[105,109,110]
**Na**	0.39–6.43	[110]
**K**	13.04–182.0	[105,110]
**Ca**	0.01–28.99	[105,109,110]
**Mg**	1.33–5.47	[105,110]
**Fe**	0.02–2.11	
**Zn**	0.02–0.067	
**Mn**	0.01–0.03	[110]
**Cu**	0.00021–0.00029	
**Se**	0.00016–0.00023	[105]

C16:0 (palmitic acid); C18:2n6c (Linoleic acid); C18:3n3 (α-Linolenic acid); C18:1n9c (oleic acid); C18:0 (stearic acid); C20:1 (eicosenoic acid); C22:1n9 (erucic acid); P—Phosphorus; Na—Sodium; K—Potassium; Ca—Calcium; Mg—Magnesium; Fe—Iron; Zn—Zinc; Mn—Manganese; Cu—Copper; Se—Selenium.

**Table 4:** Carotenoids, phenolic and other bioactive compounds of *Brassica oleracea* L. var. *italica*.

Carotenoids (mg/100 g dw)		References
**β-carotene**	9.06	[109]
**Lutein**	0.6795	
**Total Carotenoids**	46	[105]
**Chlorophylls (mg/100 g dw)**		
**C** **hlorophyll a**	14.37–447.79	[105,108,111]
**Chlorophyll b**	2.22–78.09	
**Total chlorophylls**	16.58–525.88	
**Vitamins (mg/100 g dw)**		
**Total ascorbic acid**	95	[105]
**Phenolic compounds (mg/100 g dw)**		
**Kaempherol-3-*O*-sophoroside**	0.37–3.94	
**3-Caffeoyl quinic acid**	2.50–11.51	[98]
**5-Cafeoyl quinic acid**	1.70–2.44	
**Sinapic acid**	2.13–5.43	
**Sinapic acid**	1.289	
**Neochlorogenic acid**	1.162	
**Chlorogenic acid**	1.209	
**1,2** **-Diferuloylgentiob**	2.597	
**1-Sinapoyl-2-feruloylgentiob**	8.684	
**1,2-Disinapoylgentiob**	6.198	[110]
**1,2** **′** **-Disinapoyl-2-feruloylgentiob**	1.917	
**1,2,2** **′** **-Trisinapoylgentiob**	3.191	
**K-3,7-*O*-digluc**	6.032	
**K-3-*O*-gluc-7-*O*-sophor**	1.248	
**K-3-*O*-sophorotri**	9.278	
**K-3-*O*-sophor-7-*O*-sophor**	11.126	
**K-3-*O*-feruloyldigluc-7-*O*-gluc**	2.913	
**Total polyphenols**	7.45–25.04	[100]
**Proanthocyanidins**	125	[105]
**Antioxidant activity (mg/100 g dw)**		
**DPPH**	40.81–181.49	[100,116]
**FRSA**	65.6	[117]
**Total phenolic**	94.65–1310.00	[98,100]
**Total polyphenols**	553.20–1502.00	[105,117]
**Total flavanols**	19.0	[105]
**Total phenolic acids**	389	
**Antitumor activity (GI_50_ µg/mL)**		
**U251**	61.7–>250	[119]
**MCF-7**	68.1–>250	
**786-0**	12.0–>250	
**NCI-H460**	94.9–>250	
**HT29**	178.1–>250	
**HaCaT**	58.4–>250	
**Glucosinolates (mg/100 g dw)**		
**Glucoraphanin**	0.08–6.00	[99,105,109,111,112,113,114]
**3-Methylsulfinylpropyl**	2.377	[115]
**4-Methylsulfinylbutyl**	4.769	
**Total AGS**	0.08–1.52	[111,112,114]
**Total IGS**	0.17–6.54	
**Total Glucosinolate**	0.75–9.12	[108,112,113,114,115]

DPPH—2,2-diphenyl-1-picrylhydrazyl; FRSA—Free Radical Scavenging Assay; U251—Human glioblastoma; MCF-7—breast cancer; 786-0—renal cancer; NCI-H460—lung carcinoma; HT29—colorectal adenocarcinoma; HaCaT—aneuploid immortal keratinocyte; AGS—Aliphatic glucosinolate; IGS—Indole glucosinolate.

**Table 5:** Energetic value, proximate composition, and free sugars of *Lactuca sativa* L.

Proximate Composition (g/100 g fw)		References
**Moisture (%)**	91.60–96.10	[135,136]
**Protein**	0.004–1.90	[135,136,137,138,139,140,141,142]
**Crude fat**	0.20–0.49	[135,136,138]
**Ash**	0.88	[135]
**Carbohydrates**	0.83	[136]
**Dietary fibre**	1.18	
**Energy (Kcal/100 g fw)**	11.5	[136]
**Fatty acids (%)**		
**Palmitic (C16:0)**	14.25–16.77	[143]
**Linoleic (C18:2n6c)**	13.12–17.87	
**α** **-Linolenic (C18:3n3)**	56.17–64.44	
**SFA (saturated fatty acids)**	18.75–23.03	
**MUFA (Monounsaturated fatty acids)**	8.97–3.34	
**PUFA (Polyunsaturated fatty acids)**	73.87–77.94	
**Macro and micro elements (mg/100 g fw)**		
**C**	5.89–5.93	[129]
**N**	0.28–5.56	[129,144,148]
**Mg**	0.047–27.4	[129,139,144,145,146,147,148,149]
**P**	0.033–29.5	[129,139,144,145,146,148,149]
**Ca**	0.083–26.6	[129,139,144,145,146,147,148,149]
**Zn**	0.0003–101.6	[129,144,145,146,147,148]

SFA—Saturated fatty acids; MUFA—Monounsaturated fatty acids; PUFA—Polyunsaturated fatty acids. C—Carbon; N—Nitrogen; Mg—Magnesium; P—Phosphorus; Ca—Calcium; Zn—Zinc. For uniformity reasons and when possible, values were converted from a dry weight basis or other units presented in the original source to a fresh weight basis.

**Table 6:** Carotenoids, vitamins, phenolic and bioactive compounds of *Lactuca sativa* L.

Carotenoids (mg/100 g fw)		References
**β-carotene**	0.51–30.61	[144,149]
**Carotenoids**	0.05–0.46	[142,143]
**Clorophylls (mg/100 g fw)**		
**Chlorophyll *a***	0.92–27.40	[142,144]
**Chlorophyll *b***	0.33–11.08	[142,144,148]
**Vitamins (mg/100 g fw)**		
**Total ascorbic acid**	0.44–19.4	[136,142,150,151,152,153]
**Vitamin A**	59	[136]
**Vitamin B1**	0.03	
**Vitamin B2**	0.04	
**Vitamin E**	0.35	
**Phenolic compounds (mg/100 g fw)**		
**Chicoric acid**	0.022–4.249	[129,131,145,149,150]
**Quercetin**	0.04–5.23	[129,131,144]
**Isohamnetin**	1.77–6.17	[144]
**Caffeoyl-*meso*-tartaric acid**	0.11–1.92	[149]
**Total flavonoids**	1.44–6.00	[141,150]
**Total Phenolics**	0.001–18.70	[129,141,149,150,152]
**Total Phenols**	0.001–25.500	[129,144,151]
**Anthocyanin Phenolic Compounds (mg/100 g fw)**		
**Anthocyanin**	0.001–16.0	[141,142,144,149]
**Antioxidant activity (mg/100 g fw)**		
**DPPH**	0.003–54.760	[134,143,144]
**ABTS**	0.005–6.05	[134,143,144,145]
**FRAP**	15.590–127.570	[144,145]
**AA**	22.30–96.90	[139]

DPPH—free-radical scavenging activity; ABTS—2,2′-azinobis-(3-ethylbenzothiazoline-6-sulfonate; FRAP—ferric reducing ability of plasma; AA—Total ascorbic acid. For uniformity reasons and when possible, values were converted from a dry weight basis or other units presented in the original source to a fresh weight basis.

## References

[1] Funami T., Nakauma M. (2022). Instrumental Food Texture Evaluation in Relation to Human Perception. Food Hydrocoll..

[2] Kusmayadi A., Leong Y.K., Yen H.W., Huang C.Y., Chang J.S. (2021). Microalgae as Sustainable Food and Feed Sources for Animals and Humans–Biotechnological and Environmental Aspects. Chemosphere.

[3] Bailey C., Prichard I., Drummond C., Drummond M. (2022). Australian Adolescents’ Beliefs and Perceptions towards Healthy Eating from a Symbolic and Moral Perspective: A Qualitative Study. Appetite.

[4] Mullins A.M., McRae A.E., Ansah R.M., Johnson S.B., Flessa S.J., Thornton R.L. (2022). Healthy Eating Value Systems Among Supplemental Nutrition Assistance Program Participants: A Qualitative Study. Acad. Pediatrics.

[5] Costa J. (2020). Alimentação Sustentável: Alguns Fundamentos Para Reflexão Sustainable Food: Some Fundamentals for Reflection. AdolesCiênc. Rev. Júnior Educ..

[6] Jhariya M.K., Banerjee A., Meena R.S., Yadav D.K. (2019). Agriculture, Forestry and Environmental Sustainability: A Way Forward. Sustainable Agriculture, Forest and Environmental Management.

[7] Ajila C.M., Brar S.K., Verma M., Prasada Rao U.J.S. (2012). Sustainable Solutions for Agro Processing Waste Management: An Overview. Environmental Protection Strategies for Sustainable Development.

[8] Dilucia F., Lacivita V., Conte A., del Nobile M.A. (2020). Sustainable Use of Fruit and Vegetable By-Products to Enhance Food Packaging Performance. Foods.

[9] European Commission An EU Action Plan for the Circular Economy. https://eur-lex.europa.eu/resource.

[10] Food and Agriculture Organization of the United Nations (2019). Moving forward on Food Loss and Waste Reduction Food and Agriculture. State Food Agriculture.

[11] Esparza I., Jiménez-Moreno N., Bimbela F., Ancín-Azpilicueta C., Gandía L.M. (2020). Fruit and Vegetable Waste Management: Conventional and Emerging Approaches. J. Environ. Manag..

[12] Maluf R.S., Menezes F. (2002). Caderno ‘Segurança Alimentar’. http://docplayer.com.br/423030-Caderno-seguranca-alimentar.html.

[13] Cavalli S.B. (2001). Food Safety: The Approach to Transgenic Foods. Rev. Nutr..

[14] Barling D., Fanzo J. (2018). Volume Three Advances in Food Security and Sustainability.

[15] Raigond P., Singh B., Som D., Kumar S. (2020). Potato: Nutrition and Food Security.

[16] FAO Global Food Losses and Food Waste–Extent, Causes and Prevention. SAVE FOOD: An Initiative on Food Loss and Waste Reduction. https://www.fao.org/3/i2697e/i2697e.pdf.

[17] (2005). Food and Agriculture Organization of the United Nations Trade Reforms and Food Security: Conceptualizing the Linkages.

[18] Foley J.A., Ramankutty N., Brauman K.A., Cassidy E.S., Gerber J.S., Johnston M., Mueller N.D., O’Connell C., Ray D.K., West P.C. (2011). Solutions for a Cultivated Planet. Nature.

[19] Shafiee-Jood M., Cai X. (2016). Reducing Food Loss and Waste to Enhance Food Security and Environmental Sustainability. Environ. Sci. Technol..

[20] Nordhagen S., Lambertini E., DeWaal C.S., McClafferty B., Neufeld L.M. (2022). Integrating Nutrition and Food Safety in Food Systems Policy and Programming. Glob. Food Secur..

[21] Parfitt J., Croker T., Brockhaus A. (2021). Global Food Loss and Waste in Primary Production: A Reassessment of Its Scale and Significance. Sustainability.

[22] Cole M.B., Augustin M.A., Robertson M.J., Manners J.M. (2018). The Science of Food Security. NPJ Sci. Food.

[23] Koohafkan P., Altieri M.A., Holt Gimenez E. (2012). Green Agriculture: Foundations for Biodiverse, Resilient and Productive Agricultural Systems. Int. J. Agric. Sustain..

[24] Rivera-Ferre M.G., Ortega-Cerdà M., Baumgärtner J. (2013). Rethinking Study and Management of Agricultural Systems for Policy Design. Sustainability.

[25] Desa U.N. Transforming Our World: The 2030 Agenda for Sustainable Development|Department of Economic and Social Affairs. https://sdgs.un.org/2030agenda.

[26] Laurett R., Paço A., Mainardes E.W. (2021). Antecedents and Consequences of Sustainable Development in Agriculture and the Moderator Role of the Barriers: Proposal and Test of a Structural Model. J. Rural Stud..

[27] Laurett R., Paço A., Mainardes E.W. (2021). Sustainable Development in Agriculture and Its Antecedents, Barriers and Consequences –An Exploratory Study. Sustain. Prod. Consum..

[28] Pang J., Liu X., Huang Q. (2020). A New Quality Evaluation System of Soil and Water Conservation for Sustainable Agricultural Development. Agric. Water Manag..

[29] Panel M. (2013). Sustainable Intensification: A New Paradigm for African Agriculture London Agriculture Impact.

[30] Pretty J. (2008). Agricultural Sustainability: Concepts, Principles and Evidence. Philos. Trans. R. Soc. B Biol. Sci..

[31] Food and Agriculture Organization Key to Achieving the 2030 Agenda for Sustainable Development|Policy Support and Governance|Food and Agriculture Organization of the United Nations. https://www.fao.org/policy-support/tools-and-publications/resources-details/es/c/422261/.

[32] Pretty J. (2018). Intensification for Redesigned and Sustainable Agricultural Systems. Science.

[33] Bellon S., Penvern S. (2014). Organic Farming, Prototype for Sustainable Agricultures.

[34] Girling R., Lloyd S., Padel S., Smith J., Smith L.G., Vieweger A., Wolfe M.S. (2015). The Role of Agroecology in Sustainable Intensification.

[35] Buckwell A., Uhre A.N., Williams A., Polakova J. (2014). Sustainable Intensification of European Agriculture.

[36] Garbach K., Milder J.C., Declerck F.A.J., Montenegro De Wit M., Driscoll L., Gemmill-Herren B. (2016). Examining Multi-Functionality for Crop Yield and Ecosystem Services in Five Systems of Agroecological Intensification. Int. J. Agric. Sustain..

[37] Gurr G., Lu Z., Zheng X., Xu H., Zhu P., Chen G., Plants X.Y.-N. (2016). Multi-Country Evidence That Crop Diversification Promotes Ecological Intensification of Agriculture. Nature.

[38] (2009). The Conversion to Sustainable Agriculture. The Conversion to Sustainable Agriculture: Principles, Processes, and Practices.

[39] FAO Farmer Field School Guidance (FAO, 2016)-Pesquisa Google. www.fao.org.

[40] Pretty J. (2003). Social Capital and the Collective Management of Resources. Science.

[41] Parfitt J., Barthel M., MacNaughton S. (2010). Food Waste within Food Supply Chains: Quantification and Potential for Change to 2050. Philos. Trans. R. Soc. B Biol. Sci..

[42] Lipinski B., Hanson C., Lomax J., Kitinoja L., Waite R., Searchinger T. (2013). Reducing Food Loss and Waste.

[43] Papargyropoulou E., Lozano R., Steinberger J.K., Wright N., bin Ujang Z. (2014). The Food Waste Hierarchy as a Framework for the Management of Food Surplus and Food Waste. J. Clean. Prod..

[44] Galanakis C.M. (2020). Food Waste Recovery: Processing Technologies, Industrial Techniques, and Applications.

[45] Ambuko J. (2014). Food Losses and Waste in the Context of Sustainable Food Systems A Report by The High Level Panel of Experts on Food Security and Nutrition. A Report by the High Level Panel of Experts on Food Security and Nutrition of the Committee on World Food Security.

[46] Pfaltzgraff L.A., de Bruyn M., Cooper E.C., Budarin V., Clark J.H. (2013). Food Waste Biomass: A Resource for High-Value Chemicals. Green Chem..

[47] Teigiserova D.A., Hamelin L., Thomsen M. (2020). Towards Transparent Valorization of Food Surplus, Waste and Loss: Clarifying Definitions, Food Waste Hierarchy, and Role in the Circular Economy. Sci. Total Environ..

[48] Bellemare M.F., Çakir M., Peterson H.H., Novak L., Rudi J. (2017). On the Measurement of Food Waste. Am. J. Agric. Econ..

[49] Thyberg K.L., Tonjes D.J. (2016). Drivers of Food Waste and Their Implications for Sustainable Policy Development. Resour. Conserv. Recycl..

[50] FAO (2011). Global Food Losses and Food Waste–Extent, Causes and Prevention.

[51] Girotto F., Alibardi L., Cossu R. (2015). Food Waste Generation and Industrial Uses: A Review. Waste Manag..

[52] FAO (2014). Definitional Framework of Food Loss.

[53] Garrone P., Melacini M., Perego A. (2014). Opening the Black Box of Food Waste Reduction. Food Policy.

[54] International Food Policy Research Institute (2016). Global Food Policy Report.

[55] Glanz R., Schneider F. (2009). Causes of Food Waste Generation in Households. Proc. Sard. Margherita Di Pula.

[56] Galanakis C.M. (2012). Recovery of High Added-Value Components from Food Wastes: Conventional, Emerging Technologies and Commercialized Applications. Trends Food Sci. Technol..

[57] Misi S.N., Forster C.F. (2010). Semi-Continuous Anaerobic Co-Digestion of Agro-Wastes. Environ. Technol..

[58] De Andrade R.M.S., Silva S., da Costa C.M.S.F., Veiga M., Costa E., Ferreira M.S.L., Gonçalves E.C.B.d.A., Pintado M.E. (2020). Potential Prebiotic Effect of Fruit and Vegetable Byproducts Flour Using in Vitro Gastrointestinal Digestion. Food Res. Int..

[59] Zuorro A., Lavecchia R. (2011). Polyphenols and Energy Recovery from Spent Coffee Grounds. Chem. Eng. Trans..

[60] Varjani S., Shah A.V., Vyas S., Srivastava V.K. (2021). Processes and Prospects on Valorizing Solid Waste for the Production of Valuable Products Employing Bio-Routes: A Systematic Review. Chemosphere.

[61] Sridhar A., Ponnuchamy M., Kumar P.S., Kapoor A., Vo D.V.N., Prabhakar S. (2021). Techniques and Modeling of Polyphenol Extraction from Food: A Review. Environ. Chem. Lett..

[62] Yusuf M. (2019). Agro-Industrial Waste Materials and Their Recycled Value-Added Applications: Review. Handbook of Ecomaterials.

[63] Gowe C. (2015). Review on Potential Use of Fruit and Vegetables By-Products as A Valuable Source of Natural Food Additives. Food Sci. Qual. Manag..

[64] Gullón P., Gullón B., Romaní A., Rocchetti G., Lorenzo J.M. (2020). Smart Advanced Solvents for Bioactive Compounds Recovery from Agri-Food by-Products: A Review. Trends Food Sci. Technol..

[65] Comunian T.A., Silva M.P., Souza C.J.F. (2021). The Use of Food By-Products as a Novel for Functional Foods: Their Use as Ingredients and for the Encapsulation Process. Trends Food Sci. Technol..

[66] Encalada A.M.I., Pérez C.D., Flores S.K., Rossetti L., Fissore E.N., Rojas A.M. (2019). Antioxidant Pectin Enriched Fractions Obtained from Discarded Carrots (*Daucus carota*, L.) by Ultrasound-Enzyme Assisted Extraction. Food Chem..

[67] Keser D., Guclu G., Kelebek H., Keskin M., Soysal Y., Sekerli Y.E., Arslan A., Selli S. (2020). Characterization of Aroma and Phenolic Composition of Carrot (*Daucus carota* ‘Nantes’) Powders Obtained from Intermittent Microwave Drying Using GC–MS and LC–MS/MS. Food Bioprod. Process..

[68] Santana-Gálvez J., Pérez-Carrillo E., Velázquez-Reyes H.H., Cisneros-Zevallos L., Jacobo-Velázquez D.A. (2016). Application of Wounding Stress to Produce a Nutraceutical-Rich Carrot Powder Ingredient and Its Incorporation to Nixtamalized Corn Flour Tortillas. J. Funct. Foods.

[69] Majdoub S., El Mokni R., Aliev A.M., Piras A., Porcedda S., Hammami S. (2019). Effect of Pressure Variation on the Efficiency of Supercritical Fluid Extraction of Wild Carrot (*Daucus carota* Subsp. Maritimus) Extracts. J. Chromatogr. B Anal. Technol. Biomed. Life Sci..

[70] Banwo K., Olojede A.O., Adesulu-Dahunsi A.T., Verma D.K., Thakur M., Tripathy S., Singh S., Patel A.R., Gupta A.K., Aguilar C.N. (2021). Functional Importance of Bioactive Compounds of Foods with Potential Health Benefits: A Review on Recent Trends. Food Biosci..

[71] Ma T., Tian C., Luo J., Sun X., Quan M., Zheng C., Zhan J. (2015). Influence of Technical Processing Units on the α-Carotene, β-Carotene and Lutein Contents of Carrot (*Daucus Carrot,* L.) Juice. J. Funct. Foods.

[72] Silva F.A., Queiroga R.d.C.R.d.E., de Souza E.L., Voss G.B., Borges G.d.S.C., Lima M.d.S., Pintado M.M.E., Vasconcelos M.A.d.S. (2022). Incorporation of Phenolic-Rich Ingredients from Integral Valorization of Isabel Grape Improves the Nutritional, Functional and Sensory Characteristics of Probiotic Goat Milk Yogurt. Food Chem..

[73] Mohanty B.P., Mahanty A., Ganguly S., Mitra T., Karunakaran D., Anandan R. (2019). Nutritional Composition of Food Fishes and Their Importance in Providing Food and Nutritional Security. Food Chem..

[74] Boadi N.O., Badu M., Kortei N.K., Saah S.A., Annor B., Mensah M.B., Okyere H., Fiebor A. (2021). Nutritional Composition and Antioxidant Properties of Three Varieties of Carrot (*Daucus carota*). Sci. Afr..

[75] Agirman B., Settanni L., Erten H. (2021). Effect of Different Mineral Salt Mixtures and Dough Extraction Procedure on the Physical, Chemical and Microbiological Composition of Şalgam: A Black Carrot Fermented Beverage. Food Chem..

[76] Bonasia A., Conversa G., Lazzizera C., Gambacorta G., Elia A. (2021). Morpho-Biometrical, Nutritional and Phytochemical Characterization of Carrot Landraces from Puglia Region (Southern Italy). Sustainability.

[77] Kohajdová Z., Karovičová J., Jurasová M. (2012). Influence of Carrot Pomace Powder on the Rheological Characteristics of Wheat Flour Dough and on Wheat Rolls Quality. Acta Sci. Pol. Technol. Aliment..

[78] Mizgier P., Kucharska A.Z., Sokół-Łetowska A., Kolniak-Ostek J., Kidoń M., Fecka I. (2016). Characterization of Phenolic Compounds and Antioxidant and Anti-Inflammatory Properties of Red Cabbage and Purple Carrot Extracts. J. Funct. Foods.

[79] Widaningrum, Flanagan B.M., Williams B.A., Sonni F., Mikkelsen D., Gidley M.J. (2020). Fruit and Vegetable Insoluble Dietary Fibre in Vitro Fermentation Characteristics Depend on Cell Wall Type. Bioact. Carbohydr. Diet. Fibre.

[80] Juvonen R., Honkapää K., Maina N.H., Shi Q., Viljanen K., Maaheimo H., Virkki L., Tenkanen M., Lantto R. (2015). The Impact of Fermentation with Exopolysaccharide Producing Lactic Acid Bacteria on Rheological, Chemical and Sensory Properties of Pureed Carrots (*Daucus carota*, L.). Int. J. Food Microbiol..

[81] Marszałek K., Krzyżanowska J., Woźniak, Skąpska S. (2016). Kinetic Modelling of Tissue Enzymes Inactivation and Degradation of Pigments and Polyphenols in Cloudy Carrot and Celery Juices under Supercritical Carbon Dioxide. J. Supercrit. Fluids.

[82] Stinco C.M., Szczepańska J., Marszałek K., Pinto C.A., Inácio R.S., Mapelli-Brahm P., Barba F.J., Lorenzo J.M., Saraiva J.A., Meléndez-Martínez A.J. (2019). Effect of High-Pressure Processing on Carotenoids Profile, Colour, Microbial and Enzymatic Stability of Cloudy Carrot Juice. Food Chem..

[83] Uzel R.A. (2017). A Practical Method for Isolation of Phenolic Compounds from Black Carrot Utilizing Pressurized Water Extraction with In-Site Particle Generation in Hot Air Assistance. J. Supercrit. Fluids.

[84] Zaccari F., Cabrera M.C., Ramos A., Saadoun A. (2015). In Vitro Bioaccessibility of β-Carotene, Ca, Mg and Zn in Landrace Carrots (*Daucus carota*, L.). Food Chem..

[85] Yasuda A., Kuraya E., Touyama A., Higa O., Hokamoto K., Itoh S. (2017). Underwater Shockwave Pretreatment Process for Improving Carotenoid Content and Yield of Extracted Carrot (*Daucus carota*, L.) Juice. J. Food Eng..

[86] Phan M.A.T., Bucknall M.P., Arcot J. (2019). Co-Ingestion of Red Cabbage with Cherry Tomato Enhances Digestive Bioaccessibility of Anthocyanins but Decreases Carotenoid Bioaccessibility after Simulated in Vitro Gastro-Intestinal Digestion. Food Chem..

[87] Pace B., Capotorto I., Cefola M., Minasi P., Montemurro N., Carbone V. (2020). Evaluation of Quality, Phenolic and Carotenoid Composition of Fresh-Cut Purple Polignano Carrots Stored in Modified Atmosphere. J. Food Compos. Anal..

[88] Ranjitha K., Sudhakar Rao D.V., Shivashankara K.S., Oberoi H.S., Roy T.K., Bharathamma H. (2017). Shelf-Life Extension and Quality Retention in Fresh-Cut Carrots Coated with Pectin. Innov. Food Sci. Emerg. Technol..

[89] Smeriglio A., Denaro M., Barreca D., D’Angelo V., Germanò M.P., Trombetta D. (2018). Polyphenolic Profile and Biological Activities of Black Carrot Crude Extract (*Daucus carota*, L. Ssp. Sativus Var. Atrorubens Alef.). Fitoterapia.

[90] Martínez-Flores H.E., Garnica-Romo M.G., Bermúdez-Aguirre D., Pokhrel P.R., Barbosa-Cánovas G.V. (2015). Physico-Chemical Parameters, Bioactive Compounds and Microbial Quality of Thermo-Sonicated Carrot Juice during Storage. Food Chem..

[91] González-Peña M.A., Lozada-Ramírez J.D., Ortega-Regules A.E. (2021). Carotenoids from Mamey (*Pouteria sapota*) and Carrot (*Daucus carota*) Increase the Oxidative Stress Resistance of *Caenorhabditis Elegans*. Biochem. Biophys. Rep..

[92] Kaur G.J., Orsat V., Singh A. (2020). Challenges and Potential Solutions to Utilization of Carrot Rejects and Waste in Food Processing. Br. Food J..

[93] Ahmad T., Cawood M., Iqbal Q., Ariño A., Batool A., Sabir Tariq R.M., Azam M., Akhtar S. (2019). Phytochemicals in *Daucus carota* and Their Health Benefits—Review Article. Foods.

[94] Sánchez-Rangel J.C., Benavides J., Jacobo-Velázquez D.A. (2021). Valorization of Carrot Pomace: UVC Induced Accumulation of Antioxidant Phenolic Compounds. Appl. Sci..

[95] Chiboub W., Sassi A.B., Amina C.M., Souilem F., el Ayeb A., Djlassi B., Ascrizzi R., Flamini G., Harzallah-Skhiri F. (2019). Valorization of the Green Waste from Two Varieties of Fennel and Carrot Cultivated in Tunisia by Identification of the Phytochemical Profile and Evaluation of the Antimicrobial Activities of Their Essentials Oils. Chem. Biodivers..

[96] Campas-Baypoli O.N., Snchez-Machado D.I., Bueno-Solano C., Núñez-Gastélum J.A., Reyes-Moreno C., López-Cervantes J. (2009). Biochemical Composition and Physicochemical Properties of Broccoli Flours. Int. J. Food Sci. Nutr..

[97] Xu D., Zuo J., Fang Y., Yan Z., Shi J., Gao L., Wang Q., Jiang A. (2021). Effect of Folic Acid on the Postharvest Physiology of Broccoli during Storage. Food Chem..

[98] Jin P., Yao D., Xu F., Wang H., Zheng Y. (2015). Effect of Light on Quality and Bioactive Compounds in Postharvest Broccoli Florets. Food Chem..

[99] Sakr M.T., Ibrahim H.M., ElAwady A.E., AboELMakarm A.A. (2021). Growth, Yield and Biochemical Constituents as Well as Post-Harvest Quality of Water-Stressed Broccoli (*Brassica oleraceae*, L. Var. Italica) as Affected by Certain Biomodulators. Sci. Hortic..

[100] Thomas M., Badr A., Desjardins Y., Gosselin A., Angers P. (2018). Characterization of Industrial Broccoli Discards (*Brassica oleracea* Var. Italica) for Their Glucosinolate, Polyphenol and Flavonoid Contents Using UPLC MS/MS and Spectrophotometric Methods. Food Chem..

[101] Drabińska N., Ciska E., Szmatowicz B., Krupa-Kozak U. (2018). Broccoli By-Products Improve the Nutraceutical Potential of Gluten-Free Mini Sponge Cakes. Food Chem..

[102] Hwang J.H., Lim S. (2015). Bin Antioxidant and Anticancer Activities of Broccoli By-Products from Different Cultivars and Maturity Stages at Harvest. Prev. Nutr. Food Sci..

[103] Xu J., Zhang Y., Wang W., Li Y. (2020). Advanced Properties of Gluten-Free Cookies, Cakes, and Crackers: A Review. Trends Food Sci. Technol..

[104] Shi M., Ying D.Y., Ye J.H., Sanguansri L., Augustin M.A. (2020). Broccoli Byproducts for Protection and Co-Delivery of EGCG and Tuna Oil. Food Chem..

[105] Vale A.P., Santos J., Brito N.V., Peixoto V., Carvalho R., Rosa E., Oliveira M.B.P.P. (2015). Light Influence in the Nutritional Composition of *Brassica oleracea* Sprouts. Food Chem..

[106] Ferreira S.S., Passos C.P., Cardoso S.M., Wessel D.F., Coimbra M.A. (2018). Microwave Assisted Dehydration of Broccoli By-Products and Simultaneous Extraction of Bioactive Compounds. Food Chem..

[107] Šamec D., Pavlović I., Radojčić Redovniković I., Salopek-Sondi B. (2018). Comparative Analysis of Phytochemicals and Activity of Endogenous Enzymes Associated with Their Stability, Bioavailability and Food Quality in Five Brassicaceae Sprouts. Food Chem..

[108] Shi M., Hlaing M.M., Ying D.Y., Ye J.H., Sanguansri L., Augustin M.A. (2019). New Food Ingredients from Broccoli By-Products: Physical, Chemical and Technological Properties. Int. J. Food Sci. Technol..

[109] Hu C.H., Wang D.G., Pan H.Y., Zheng W.B., Zuo A.Y., Liu J.X. (2012). Effects of Broccoli Stem and Leaf Meal on Broiler Performance, Skin Pigmentation, Antioxidant Function, and Meat Quality. Poult. Sci..

[110] Liu M., Zhang L., Ser S.L., Cumming J.R., Ku K.M. (2018). Comparative Phytonutrient Analysis of Broccoli By-Products: The Potentials for Broccoli by-Product Utilization. Molecules.

[111] Fernández-León M.F., Fernández-León A.M., Lozano M., Ayuso M.C., González-Gómez D. (2013). Altered Commercial Controlled Atmosphere Storage Conditions for “Parhenon” Broccoli Plants (*Brassica oleracea*, L. Var. Italica). Influence on the Outer Quality Parameters and on the Health-Promoting Compounds. LWT Food Sci. Technol..

[112] Flores P., Pedreño M.A., Almagro L., Hernández V., Fenoll J., Hellín P. (2021). Increasing Nutritional Value of Broccoli with Seaweed Extract and Trilinolein. J. Food Compos. Anal..

[113] Cai C., Miao H., Qian H., Yao L., Wang B., Wang Q. (2016). Effects of Industrial Pre-Freezing Processing and Freezing Handling on Glucosinolates and Antioxidant Attributes in broccoli florets. Food Chem..

[114] Chiu Y.C., Matak K., Ku K.M. (2019). Methyl Jasmonate Treated Broccoli: Impact on the Production of Glucosinolates and Consumer Preferences. Food Chem..

[115] Li Z., Zheng S., Liu Y., Fang Z., Yang L., Zhuang M., Zhang Y., Lv H., Wang Y., Xu D. (2021). Characterization of Glucosinolates in 80 Broccoli Genotypes and Different Organs Using UHPLC-Triple-TOF-MS Method. Food Chem..

[116] Rios J.J., Pascual J.A., Guillen M., Lopez-Martinez A., Carvajal M. (2021). Influence of Foliar Methyl-Jasmonate Biostimulation on Exudation of Glucosinolates and Their Effect on Root Pathogens of Broccoli Plants under Salinity Condition. Sci. Hortic..

[117] Mewis I., Schreiner M., Nguyen C.N., Krumbein A., Ulrichs C., Lohse M., Zrenner R. (2012). UV-B Irradiation Changes Specifically the Secondary Metabolite Profile in Broccoli Sprouts: Induced Signaling Overlaps with Defense Response to Biotic Stressors. Plant Cell Physiol..

[118] Kabir F., Tow W.W., Hamauzu Y., Katayama S., Tanaka S., Nakamura S. (2015). Antioxidant and Cytoprotective Activities of Extracts Prepared from Fruit and Vegetable Wastes and By-Products. Food Chem..

[119] Mahn A. (2017). Modelling of the Effect of Selenium Fertilization on the Content of Bioactive Compounds in Broccoli Heads. Food Chem..

[120] Bachiega P., Salgado J.M., De Carvalho J.E., Ruiz A.L.T.G., Schwarz K., Tezotto T., Morzelle M.C. (2016). Antioxidant and Antiproliferative Activities in Different Maturation Stages of Broccoli (*Brassica oleracea* Italica) Biofortified with Selenium. Food Chem..

[121] de Evan T., Vintimilla A., Marcos C.N., Ranilla M.J., Carro M.D. (2019). Evaluation of Brassica Vegetables as Potential Feed for Ruminants. Animals.

[122] Landin-Sandoval V.J., Mendoza-Castillo D.I., Bonilla-Petriciolet A., Aguayo-Villarreal I.A., Reynel-Avila H.E., Gonzalez-Ponce H.A. (2020). Valorization of Agri-Food Industry Wastes to Prepare Adsorbents for Heavy Metal Removal from Water. J. Environ. Chem. Eng..

[123] de Evan T., Marcos C.N., Ranilla M.J., Carro M.D. (2020). In Vitro and in Situ Evaluation of Broccoli Wastes as Potential Feed for Ruminants. Animals.

[124] Poultry World Broccoli: Antimicrobial and Antioxidant Benefits in Broilers-Poultry World. https://www.poultryworld.net/health-nutrition/broccoli-antimicrobial-and-antioxidant-benefits-in-broilers/.

[125] Sanz-Puig M., Pina-Pérez M.C., Criado M.N., Rodrigo D., Martínez-López A. (2015). Antimicrobial Potential of Cauliflower, Broccoli, and Okara Byproducts against Foodborne Bacteria. Foodborne Pathog. Dis..

[126] Galieni A., Di Mattia C., De Gregorio M., Speca S., Mastrocola D., Pisante M., Stagnari F. (2015). Effects of Nutrient Deficiency and Abiotic Environmental Stresses on Yield, Phenolic Compounds and Antiradical Activity in lettuce (*Lactuca sativa*, L.). Sci. Hortic..

[127] Matraszek R., Hawrylak-Nowak B., Chwil S., Chwil M. (2016). Macroelemental Composition of Cadmium Stressed Lettuce Plants Grown under Conditions of Intensive Sulphur Nutrition. J. Environ. Manag..

[128] Pinto E., Almeida A.A., Aguiar A.A.R.M., Ferreira I.M. (2014). Changes in Macrominerals, Trace Elements and Pigments Content during Lettuce (*Lactuca sativa*, L.) Growth: Influence of Soil Composition. Food Chem..

[129] Sofo A., Lundegårdh B., Mårtensson A., Manfra M., Pepe G., Sommella E., De Nisco M., Tenore G.C., Campiglia P., Scopa A. (2016). Different Agronomic and Fertilization Systems Affect Polyphenolic Profile, Antioxidant Capacity and Mineral Composition of Lettuce. Sci. Hortic..

[130] Ouyang Z., Tian J., Yan X., Shen H. (2020). Effects of Different Concentrations of Dissolved Oxygen or Temperatures on the Growth, Photosynthesis, Yield and Quality of Lettuce. Agric. Water Manag..

[131] Pepe G., Sommella E., Manfra M., De Nisco M., Tenore G.C., Scopa A., Sofo A., Marzocco S., Adesso S., Novellino T. (2015). Evaluation of Anti-Inflammatory Activity and Fast UHPLC-DAD-IT-TOF Profiling of Polyphenolic Compounds Extracted from Green Lettuce (*Lactuca sativa*, L.; Var. Maravilla de Verano). Food Chem..

[132] Chadwick M., Gawthrop F., Michelmore R.W., Wagstaff C., Methven L. (2016). Perception of Bitterness, Sweetness and Liking of Different Genotypes of Lettuce. Food Chem..

[133] Choi H.S., Han J.Y., Choi Y.E. (2020). Identification of Triterpenes and Functional Characterization of Oxidosqualene Cyclases Involved in Triterpene Biosynthesis in Lettuce (*Lactuca sativa*). Plant Sci..

[134] Harsha S.N., Anilakumar K.R., Mithila M.V. (2013). Antioxidant Properties of *Lactuca sativa* Leaf Extract Involved in the Protection of Biomolecules. Biomed. Prev. Nutr..

[135] Ramos-Sotelo H., Valdez-Ortiz Á., Germán-Báez L.J., Fierro-Sañudo J.F., León-Cañedo J.A., Alarcón-Silvas S.G., Reyes-Moreno C., Páez-Osuna F. (2019). Quality of Lettuce *Lactuca sativa* (Var. Tropicana M1) Grown with Two Low-Salinity Shrimp Effluents. Food Chem. X.

[136] Yoshida T., Sakuma K., Kumagai H. (2014). Nutritional and Taste Characteristics of Low-Potassium Lettuce Developed for Patients with Chronic Kidney Diseases. Hong Kong J. Nephrol..

[137] Baslam M., Morales F., Garmendia I., Goicoechea N. (2013). Nutritional Quality of Outer and Inner Leaves of Green and Red Pigmented Lettuces (*Lactuca sativa*, L.) Consumed as Salads. Sci. Hortic..

[138] Kopeć A., Piatkowska E., Biezanowska-Kopeć R., Pysz M., Koronowicz A., Kapusta-Duch J., Smoleń S., Rakoczy R., Skoczylas Ł., Leszczyńska T. (2015). Effect of Lettuce Biofortified with Iodine by Soil Fertilization on Iodine Concentration in Various Tissues and Selected Biochemical Parameters in Serum of Wistar Rats. J. Funct. Foods.

[139] Colonna E., Rouphael Y., Barbieri G., De Pascale S. (2016). Nutritional Quality of Ten Leafy Vegetables Harvested at Two Light Intensities. Food Chem..

[140] Bian Z., Cheng R., Wang Y., Yang Q., Lu C. (2018). Effect of Green Light on Nitrate Reduction and Edible Quality of Hydroponically Grown Lettuce (*Lactuca sativa* L.) under Short-Term Continuous Light from Red and Blue Light-Emitting Diodes. Environ. Exp. Bot..

[141] Chen Y., Li T., Yang Q., Zhang Y., Zou J., Bian Z., Wen X. (2019). UVA Radiation Is Beneficial for Yield and Quality of Indoor Cultivated Lettuce. Front. Plant Sci..

[142] He R., Zhang Y., Song S., Su W., Hao Y., Liu H. (2021). UV-A and FR Irradiation Improves Growth and Nutritional Properties of Lettuce Grown in an Artificial Light Plant Factory. Food Chem..

[143] Kim D.E., Shang X., Assefa A.D., Keum Y.S., Saini R.K. (2018). Metabolite Profiling of Green, Green/Red, and Red Lettuce Cultivars: Variation in Health Beneficial Compounds and Antioxidant Potential. Food Res. Int..

[144] Mampholo B.M., Maboko M.M., Soundy P., Sivakumar D. (2016). Phytochemicals and Overall Quality of Leafy Lettuce (*Lactuca sativa* L.) Varieties Grown in Closed Hydroponic System. J. Food Qual..

[145] Managa M.G., Tinyani P.P., Senyolo G.M., Soundy P., Sultanbawa Y., Sivakumar D. (2018). Impact of Transportation, Storage, and Retail Shelf Conditions on Lettuce Quality and Phytonutrients Losses in the Supply Chain. Food Sci. Nutr..

[146] Qin X.X., Zhang M.Y., Han Y.Y., Hao J.H., Liu C.J., Fan S.X. (2018). Beneficial Phytochemicals with Anti-Tumor Potential Revealed through Metabolic Profiling of New Red Pigmented Lettuces (*Lactuca sativa,* L.). Int. J. Mol. Sci..

[147] Hattab S., Bougattass I., Hassine R., Dridi-Al-Mohandes B. (2019). Metals and Micronutrients in Some Edible Crops and Their Cultivation Soils in Eastern-Central Region of Tunisia: A Comparison between Organic and Conventional Farming. Food Chem..

[148] Turan V. (2019). Confident Performance of Chitosan and Pistachio Shell Biochar on Reducing Ni Bioavailability in Soil and Plant plus Improved the Soil Enzymatic Activities, Antioxidant Defense System and Nutritional Quality of Lettuce. Ecotoxicol. Environ. Saf..

[149] El-Nakhel C., Petropoulos S.A., Pannico A., Kyriacou M.C., Giordano M., Colla G., Troise A.D., Vitaglione P., De Pascale S., Rouphael Y. (2020). The Bioactive Profile of Lettuce Produced in a Closed Soilless System as Configured by Combinatorial Effects of Genotype and Macrocation Supply Composition. Food Chem..

[150] Santos F.T., Goufo P., Santos C., Botelho D., Fonseca J., Queirós A., Costa M.S.S.M., Trindade H. (2016). Comparison of Five Agro-Industrial Waste-Based Composts as Growing Media for Lettuce: Effect on Yield, Phenolic Compounds and Vitamin, C. Food Chem..

[151] Wieczyńska J., Cavoski I. (2018). Antimicrobial, Antioxidant and Sensory Features of Eugenol, Carvacrol and Trans-Anethole in Active Packaging for Organic Ready-to-Eat Iceberg Lettuce. Food Chem..

[152] Riga P., Benedicto L., Gil-Izquierdo Á., Collado-González J., Ferreres F., Medina S. (2019). Diffuse Light Affects the Contents of Vitamin C, Phenolic Compounds and Free Amino Acids in Lettuce Plants. Food Chem..

[153] Ku Y.G., Bae J.H., Martinez-Ayala A.L., Vearasilp S., Namieśnik J., Pasko P., Katrich E., Gorinstein S. (2017). Efficient Three-Dimensional Fluorescence Measurements for Characterization of Binding Properties in Some Plants. Sens. Actuators B Chem..

[154] Nasrin T.A.A., Matin M.A. (2017). Valorization of Vegetable Wastes. Food Processing By-Products and their Utilization.

[155] Llorach R., Tomás-Barberán F.A., Ferreres F. (2004). Lettuce and Chicory Byproducts as a Source of Antioxidant Phenolic Extracts. J. Agric. Food Chem..

[156] Russ W., Meyer-Pittroff R. (2004). Utilizing Waste Products from the Food Production and Processing Industries. Crit. Rev. Food Sci. Nutr..

[157] Murugan K., Ramasamy K. (2013). Environmental Concerns and Sustainable Development. Valorization of Food Processing By-Products.

[158] Wadhwa M., Bakshi M.P.S., Makkar H.P.S. (2015). Waste to Worth: Fruit Wastes and by-Products as Animal Feed. CAB Rev. Perspect. Agric. Vet. Sci. Nutr. Nat. Resour..

[159] Angulo J., Mahecha L., Yepes S.A., Yepes A.M., Bustamante G., Jaramillo H., Valencia E., Villamil T., Gallo J. (2012). Nutritional Evaluation of Fruit and Vegetable Waste as Feedstuff for Diets of Lactating Holstein Cows. J. Environ. Manag..

[160] García A.J., Esteban M.B., Márquez M.C., Ramos P. (2005). Biodegradable Municipal Solid Waste: Characterization and Potential Use as Animal Feedstuffs. Waste Manag..

[161] Pham T.P.T., Kaushik R., Parshetti G.K., Mahmood R., Balasubramanian R. (2015). Food Waste-to-Energy Conversion Technologies: Current Status and Future Directions. Waste Manag..

[162] Shen F., Yuan H., Pang Y., Chen S., Zhu B., Zou D., Liu Y., Ma J., Yu L., Li X. (2013). Performances of Anaerobic Co-Digestion of Fruit & Vegetable Waste (FVW) and Food Waste (FW): Single-Phase vs. Two-Phase. Bioresour. Technol..

[163] Iuga M., Mironeasa S. (2020). Potential of Grape Byproducts as Functional Ingredients in Baked Goods and Pasta. Compr. Rev. Food Sci. Food Saf..

[164] Wani A.A., Sogi D.S., Singh P., Khatkar B.S. (2015). Influence of Watermelon Seed Protein Concentrates on Dough Handling, Textural and Sensory Properties of Cookies. J. Food Sci. Technol..

[165] Santos E., Andrade R., Gouveia E. (2017). Utilization of the Pectin and Pulp of the Passion Fruit from Caatinga as Probiotic Food Carriers. Food Biosci..

[166] Tiwari B.K., Brennan C.S., Jaganmohan R., Surabi A., Alagusundaram K. (2011). Utilisation of Pigeon Pea (Cajanus Cajan L) Byproducts in Biscuit Manufacture. LWT Food Sci. Technol..

[167] Rosales Soto M.U., Brown K., Ross C.F. (2012). Antioxidant Activity and Consumer Acceptance of Grape Seed Flour-Containing Food Products. Int. J. Food Sci. Technol..

[168] Sagar N.A., Pareek S., Sharma S., Yahia E.M., Lobo M.G. (2018). Fruit and Vegetable Waste: Bioactive Compounds, Their Extraction, and Possible Utilization. Compr. Rev. Food Sci. Food Saf..

[169] Sainz R.L., Szezecinski A.C.S.F., Fontana M., Bosenbecker V.K., Ferri V.C., do Nascimento C.O. (2019). Uso de Harina de Baya de Uva En La Producción de Cookies. BIO Web Conf..

[170] Aparecida Damasceno K., Alvarenga Gonçalves C.A., dos Santos Pereira G., Lacerda Costa L., Bastianello Campagnol P.C., Leal De Almeida P., Arantes-Pereira L. (2016). Development of Cereal Bars Containing Pineapple Peel Flour (Ananas *Comosus* L. Merril). J. Food Qual..

[171] Sójka M., Kołodziejczyk K., Milala J., Abadias M., Viñas I., Guyot S., Baron A. (2015). Composition and Properties of the Polyphenolic Extracts Obtained from Industrial Plum Pomaces. J. Funct. Foods.

[172] Mieres Pitre A., Andrade A., Garca L., Londoño P. (2011). Desarrollo de Una Galleta a Partir Del Orujo de Uva Variedad Criolla Negra/Development of a Cookie from Marc Creole Black Grape Variety. Rev. An..

[173] Lohani U.C., Muthukumarappan K. (2017). Effect of Extrusion Processing Parameters on Antioxidant, Textural and Functional Properties of Hydrodynamic Cavitated Corn Flour, Sorghum Flour and Apple Pomace-Based Extrudates. J. Food Process Eng..

[174] Koca I., Tekguler B., Yilmaz V.A., Hasbay I., Koca A.F. (2018). The Use of Grape, Pomegranate and Rosehip Seed Flours in Turkish Noodle (Erişte) Production. J. Food Process. Preserv..

[175] Martínez-Hernández G.B., Castillejo N., Artés-Hernández F. (2019). Effect of Fresh–Cut Apples Fortification with Lycopene Microspheres, Revalorized from Tomato by-Products, during Shelf Life. Postharvest Biol. Technol..

[176] Ying D.Y., Hlaing M.M., Lerisson J., Pitts K., Cheng L., Sanguansri L., Augustin M.A. (2017). Physical Properties and FTIR Analysis of Rice-Oat Flour and Maize-Oat Flour Based Extruded Food Products Containing Olive Pomace. Food Res. Int..

[177] Masli M.D.P., Gu B.J., Rasco B.A., Ganjyal G.M. (2018). Fiber-Rich Food Processing Byproducts Enhance the Expansion of Cornstarch Extrudates. J. Food Sci..

[178] Bellur Nagarajaiah S., Prakash J. (2015). Nutritional Composition, Acceptability, and Shelf Stability of Carrot Pomace-Incorporated Cookies with Special Reference to Total and β-Carotene Retention. Cogent Food Agric..

[179] Costa C., Lucera A., Marinelli V., del Nobile M.A., Conte A. (2018). Influence of Different By-Products Addition on Sensory and Physicochemical Aspects of Primosale Cheese. J. Food Sci. Technol..

[180] Kampuse S., Ozola L., Straumite E., Galoburda R. (2015). Quality Parameters Of Wheat Bread Enriched With Pumpkin ( Cucurbita Moschata ) By-Products. Acta Univ. Cibiniensis. Ser. E Food Technol..

[181] Nishio N., Nakashimada Y. (2013). Manufacture of Biogas and Fertilizer from Solid Food Wastes by Means of Anaerobic Digestion. Food Industry Wastes.

[182] Pesta G. (2007). Anaerobic Digestion of Organic Residues and Wastes. Utilization of By-Products and Treatment of Waste in the Food Industry.

[183] Bres P., Beily M.E., Young B.J., Gasulla J., Butti M., Crespo D., Candal R., Komilis D. (2018). Performance of Semi-Continuous Anaerobic Co-Digestion of Poultry Manure with Fruit and Vegetable Waste and Analysis of Digestate Quality: A Bench Scale Study. Waste Manag..

[184] Ganesh K.S., Sridhar A., Vishali S. (2022). Utilization of Fruit and Vegetable Waste to Produce Value-Added Products: Conventional Utilization and Emerging Opportunities-A Review. Chemosphere.

[185] Banks C., Wang Z. (2005). Treatment of Meat Wastes. Waste Treatment in the Food Processing Industry.

[186] Shilev S., Naydenov M., Vancheva V., Aladjadjiyan A. (2007). Composting of Food and Agricultural Wastes. Utilization of By-Products and Treatment of Waste in the Food Industry.

[187] Chang J.I., Tsai J.J., Wu K.H. (2006). Composting of Vegetable Waste. Waste Manag. Res..

[188] Ghinea C., Apostol L.C., Prisacaru A.E., Leahu A. (2019). Development of a Model for Food Waste Composting. Environ. Sci. Pollut. Res..

[189] Abu Bakar A., Gawi S.N.A.S.M., Mahmood N.Z., Abdullah N. (2014). Vermicomposting of Vegetable Waste Amended with Different Sources of Agro-Industrial by-Product Using Lumbricus Rubellus. Pol. J. Environ. Stud..

[190] Deshmukh M., Deshpande R.S., Tondon G.D. (2010). Biofuel from Cellulosic Agricultural Waste. Int. J. Chem. Eng. Res..

[191] Maeda R.N., Barcelos C.A., Anna L.M.M.S., Pereira N. (2013). Cellulase Production by Penicillium Funiculosum and Its Application in the Hydrolysis of Sugar Cane Bagasse for Second Generation Ethanol Production by Fed Batch Operation. J. Biotechnol..

[192] Gosavi P., Chaudhary Y., Durve-Gupta A. (2017). Production of Biofuel from Fruits and Vegetable Wastes. Eur. J. Biotechnol. Biosci. Eur. J. Biotechnol. Biosci. Online ISSN.

[193] Sridhar A., Ponnuchamy M., Kumar P.S., Kapoor A. (2021). Food Preservation Techniques and Nanotechnology for Increased Shelf Life of Fruits, Vegetables, Beverages and Spices: A Review. Environ. Chem. Lett..

[194] Gil R.R., Ruiz B., Lozano M.S., Martín M.J., Fuente E. (2014). VOCs Removal by Adsorption onto Activated Carbons from Biocollagenic Wastes of Vegetable Tanning. Chem. Eng. J..

[195] Srivastava N.S.L., Narnaware S.L., Makwana J.P., Singh S.N., Vahora S. (2014). Investigating the Energy Use of Vegetable Market Waste by Briquetting. Renew. Energy.

[196] Raju C.A.I. (2014). Studies on Development of Fluel Briquettes for Household and Industrial Purpose. Int. J. Res. Eng. Technol..

[197] Kumar K., Yadav A.N., Kumar V., Vyas P., Dhaliwal H.S. (2017). Food Waste: A Potential Bioresource for Extraction of Nutraceuticals and Bioactive Compounds. Bioresour. Bioprocess.

[198] Swallah M.S., Sun H., Affoh R., Fu H., Yu H. (2020). Antioxidant Potential Overviews of Secondary Metabolites (Polyphenols) in Fruits. Int. J. Food Sci..

[199] Garcia-Amezquita L.E., Tejada-Ortigoza V., Serna-Saldivar S.O., Welti-Chanes J. (2018). Dietary Fiber Concentrates from Fruit and Vegetable By-Products: Processing, Modification, and Application as Functional Ingredients. Food Bioprocess Technol..

[200] Gonçalves de Moura I., Vasconcelos de Sá A., Lemos Machado Abreu A.S., Alves Machado A.V. (2017). Bioplastics from Agro-Wastes for Food Packaging Applications. Food Packaging.

[201] Dey A., Dhumal C.V., Sengupta P., Kumar A., Pramanik N.K., Alam T. (2021). Challenges and Possible Solutions to Mitigate the Problems of Single-Use Plastics Used for Packaging Food Items: A Review. J. Food Sci. Technol..

[202] Dasumiati, Saridewi N., Malik M. Food Packaging Development of Bioplastic from Basic Waste of Cassava Peel (Manihot Uttilisima) and Shrimp Shell. Proceedings of the IOP Conference Series: Materials Science and Engineering.

[203] Martínez O., Sánchez A., Font X., Barrena R. (2018). Enhancing the Bioproduction of Value-Added Aroma Compounds via Solid-State Fermentation of Sugarcane Bagasse and Sugar Beet Molasses: Operational Strategies and Scaling-up of the Process. Bioresour. Technol..

[204] Lun O.K., Wai T.B., Ling L.S. (2014). Pineapple Cannery Waste as a Potential Substrate for Microbial Biotranformation to Produce Vanillic Acid and Vanillin. Int. Food Res. J..

[205] Mantzouridou F.T., Paraskevopoulou A., Lalou S. (2015). Yeast Flavour Production by Solid State Fermentation of Orange Peel Waste. Biochem. Eng. J..

[206] Usman A.I., Aziz A.A., Noqta O.A. (2019). Application of Green Synthesis of Gold Nanoparticles: A Review. J. Teknol..

[207] Karimi M., Sadeghi R., Kokini J. (2017). Pomegranate as a Promising Opportunity in Medicine and Nanotechnology. Trends Food Sci. Technol..

[208] Jeyarani S., Vinita N.M., Puja P., Senthamilselvi S., Devan U., Velangani A.J., Biruntha M., Pugazhendhi A., Kumar P. (2020). Biomimetic Gold Nanoparticles for Its Cytotoxicity and Biocompatibility Evidenced by Fluorescence-Based Assays in Cancer (MDA-MB-231) and Non-Cancerous (HEK-293) Cells. J. Photochem. Photobiol. B Biol..

[209] Rajendran A. (2017). Antibacterial Properties and Mechanism of Gold Nanoparticles Obtained from Pergularia Daemia Leaf Extract. J. Nanomed. Res..

[210] Rajkumar P., Kailappan R., Viswanathan R., Raghavan G.S.V., Ratti C. (2007). Foam Mat Drying of Alphonso Mango Pulp. Dry Technol..

[211] Jumah R., Al-Asheh S., Banat F., Al-Zoubi K. (2005). Electroosmotic Dewatering of Tomato Paste Suspension under AC Electric Field. Dry. Technol..

[212] Díaz O., Pereira C.D., Cobos A. (2004). Functional Properties of Ovine Whey Protein Concentrates Produced by Membrane Technology after Clarification of Cheese Manufacture By-Products. Food Hydrocoll..

[213] Galanakis C.M., Tornberg E., Gekas V. (2010). A Study of the Recovery of the Dietary Fibres from Olive Mill Wastewater and the Gelling Ability of the Soluble Fibre Fraction. LWT Food Sci. Technol..

[214] Fernandes P., Cabral J.M.S. (2007). Phytosterols: Applications and Recovery Methods. Bioresour. Technol..

[215] Galanakis C.M. (2015). Separation of Functional Macromolecules and Micromolecules: From Ultrafiltration to the Border of Nanofiltration. Trends Food Sci. Technol..

[216] Galanakis C.M., Chasiotis S., Botsaris G., Gekas V. (2014). Separation and Recovery of Proteins and Sugars from Halloumi Cheese Whey. Food Res. Int..

[217] Gehring C.K., Gigliotti J.C., Moritz J.S., Tou J.C., Jaczynski J. (2011). Functional and Nutritional Characteristics of Proteins and Lipids Recovered by Isoelectric Processing of Fish By-Products and Low-Value Fish: A Review. Food Chem..

[218] Luque de Castro M.D., Priego-Capote F. (2007). Ultrasound-Assisted Crystallization (Sonocrystallization). Ultrason. Sonochem..

[219] Patel S.R., Murthy Z.V.P. (2010). Optimization of Process Parameters by Taguchi Method in the Recovery of Lactose from Whey Using Sonocrystallization. Cryst. Res. Technol..

[220] Saygi K.O., Bayram H.M., Bayram E. (2022). Green Synthesis of Silver Nanoparticles Using Artichoke Flower Petals and Application in Endodontic Dentistry. Biomass Convers. Biorefin..

[221] Farhat A., Fabiano-Tixier A.S., Maataoui M.E., Maingonnat J.F., Romdhane M., Chemat F. (2011). Microwave Steam Diffusion for Extraction of Essential Oil from Orange Peel: Kinetic Data, Extract’s Global Yield and Mechanism. Food Chem..

[222] Galanakis C.M. (2013). Emerging Technologies for the Production of Nutraceuticals from Agricultural By-Products: A Viewpoint of Opportunities and Challenges. Food Bioprod. Process..

[223] Vorobiev E., Lebovka N. (2010). Enhanced Extraction from Solid Foods and Biosuspensions by Pulsed Electrical Energy. Food Eng. Rev..

[224] Sowbhagya H.B., Chitra V.N. (2010). Enzyme-Assisted Extraction of Flavorings and Colorants from Plant Materials. Crit. Rev. Food Sci. Nutr..

[225] Panchev I.N., Kirtchev N.A., Dimitrov D.D. (2011). Possibilities for Application of Laser Ablation in Food Technologies. Innov. Food Sci. Emerg. Technol..

[226] Soto M.L., Moure A., Domínguez H., Parajó J.C. (2011). Recovery, Concentration and Purification of Phenolic Compounds by Adsorption: A Review. J. Food Eng..

[227] El-Sayed M.M.H., Chase H.A. (2011). Trends in Whey Protein Fractionation. Biotechnol. Lett..

[228] Fernández-Bolaños J., Heredia A., Rodríguez G., Rodríguez R., Guillén R., Jiménez A. (2002). Method for Obtaining Purified Hydroxytyrosol from Products and By-Products Derived from the Olive Tree. U.S. Patent.

[229] Rahmanian N., Jafari S.M., Galanakis C.M. (2014). Recovery and Removal of Phenolic Compounds from Olive Mill Wastewater. JAOCS J. Am. Oil Chem. Soc..

[230] Chen J. (1992). Partitioning and Separation of A-Lactalbumin and Fl-Lactoglobulin in PEG / Potassium Phosphate Aqueous Two-Phase Systems. J. Ferment. Bioeng..

[231] Pérez-Serradilla J.A., Luque de Castro M.D. (2011). Microwave-Assisted Extraction of Phenolic Compounds from Wine Lees and Spray-Drying of the Extract. Food Chem..

[232] Bhattacharjee S., Bhattacharjee C., Datta S. (2006). Studies on the Fractionation of β-Lactoglobulin from Casein Whey Using Ultrafiltration and Ion-Exchange Membrane Chromatography. J. Membr. Sci..

[233] Koduvayur Habeebullah S.F., Nielsen N.S., Jacobsen C. (2010). Antioxidant Activity of Potato Peel Extracts in a Fish-Rapeseed Oil Mixture and in Oil-in-Water Emulsions. JAOCS J. Am. Oil Chem. Soc..

[234] Jaeger H., Janositz A., Knorr D. (2010). The Maillard Reaction and Its Control during Food Processing. The Potential of Emerging Technologies. Pathol. Biol..

[235] McClements D.J., Rao J. (2011). Food-Grade Nanoemulsions: Formulation, Fabrication, Properties, Performance, Biological Fate, and Potential Toxicity. Crit. Rev. Food Sci. Nutr..

